# Development and validation of a harmonized memory score for multicenter Alzheimer’s disease and related dementia research

**DOI:** 10.1186/s13195-026-02100-w

**Published:** 2026-06-15

**Authors:** Mark Sanderson-Cimino, Alden L. Gross, Leslie S. Gaynor, Emily W. Paolillo, Rowan Saloner, Marilyn S. Albert, Liana G. Apostolova, Brooke Boersema, Adam L. Boxer, Bradley F. Boeve, Kaitlin B. Casaletto, Hilary W. Heuer, Leah K. Forsberg, Savannah R. Hallgarth, Valentina E. Diaz, Lindsay R. Clark, Ani Eloyan, Sarah Tomaszewski Farias, Pauline Maillard, Mitzi M. Gonzales, Dustin B. Hammers, Renaud La Joie, Yann Cobigo, Amy Wolf, Benjamin M. Hampstead, Dawn Mechanic-Hamilton, Bruce L. Miller, Gil D. Rabinovici, John M. Ringman, Lina M. D’Orazio, Howie J. Rosen, Sephira G. Ryman, Jillian L. Prestopnik, David P. Salmon, Glenn E. Smith, Charles DeCarli, Kumar B. Rajan, Lee-Way Jin, Jason D. Hinman, David K. Johnson, Danielle J. Harvey, Myriam Fornage, Maria C. Carrillo, Bradford C. Dickerson, Joel H. Kramer, Adam M. Staffaroni

**Affiliations:** 1 Edward and Pearl Fein Memory and Aging Center, UCSF Weill Institute for Neurosciences, University of California, San Francisco, San Francisco, CA 94158 USA; 2 Department of Epidemiology, Johns Hopkins Bloomberg School of Public Health, Baltimore, MD 21205 USA; 3 Department of Medicine, Division of Geriatric Medicine, Vanderbilt University Medical Center, Nashville, TN 37232 USA; 4 Vanderbilt Memory and Aging Center, Vanderbilt University Medical Center, Nashville, TN 37232 USA; 5 Department of Neurology, Johns Hopkins University School of Medicine, Baltimore, MD 21205 USA; 6 Department of Neurology, Indiana University School of Medicine, Indianapolis, IN 46202 USA; 7 Department of Neurology, Mayo Clinic, Rochester, MN 55905 USA; 8 Wisconsin Alzheimer’s Disease Research Center, University of Wisconsin School of Medicine and Public Health, Madison, WI 53726 USA; 9 Department of Biostatistics, Center for Statistical Sciences, Brown University, Providence, RI 02912 USA; 10 Department of Neurology, University of California at Davis, Sacramento, CA 95816 USA; 11 Department of Neurology, Cedars Sinai Medical Center, Los Angeles, CA 90048 USA; 12 Department of Psychiatry, University of Michigan, Ann Arbor, MI 48109 USA; 13 Department of Neurology, Perelman School of Medicine at the University of Pennsylvania, Philadelphia, PA 19104 USA; 14 Department of Neurology, Keck School of Medicine at USC, Los Angeles, CA 90033 USA; 15 Center for Memory and Aging, University of New Mexico, Albuquerque, NM 87110 USA; 16 Department of Neurosciences, University of California, San Diego, La Jolla, CA 92161 USA; 17 1Florida Alzheimer’s Disease Research Center, Gainesville, FL 32610 USA; 18 Department of Clinical and Health Psychology, University of Florida, Gainesville, FL 32603 USA; 19 Rush Institute for Healthy Aging, Rush University Medical Center, Chicago, IL 60612 USA; 20 Department of Pathology and Laboratory Medicine, University of California, Davis, CA 95817 USA; 21 Department of Neurology, University of California, Los Angeles, Los Angeles, CA 90095 USA; 22 Department of Public Health Sciences, University of California, Davis, CA 95616 USA; 23 Brown Foundation Institute of Molecular Medicine, McGovern Medical School at The University of Texas Health Science Center at Houston, Houston, TX 77030 USA; 24 Division of Medical and Scientific Relations, Alzheimer’s Association, Chicago, IL 60603 USA; 25 Department of Neurology, Massachusetts General Hospital/Harvard Medical School, Boston, MA 02129 USA

**Keywords:** Memory, Alzheimer’s Dementia and Related Disorders, Harmonization, Co-calibration, Neuropsychology, Cognition, Neurodegeneration

## Abstract

**Introduction:**

List-learning tasks (LLTs) are important for characterizing memory in Alzheimer’s disease and related dementias. However, as the Uniform Data Set Neuropsychological Battery (UDS-NB) historically did not require a specific LLT, centers have administered different LLTs. The newest UDS version (v4.0) requires one of two LLTs. To support harmonized UDS memory research, we developed a memory composite incorporating UDS memory tests and multiple LLTs.

**Methods:**

Item-banking confirmatory factor analysis was applied to develop a memory composite in a diagnostically heterogeneous sample (*n* = 8716) that completed the UDS-NB and one of five LLTs. Construct validity was evaluated through associations with demographics, disease severity, cognitive tasks, brain volume, amyloid/tau PET positivity, and plasma phosphorylated tau. Analyses were replicated in a racially/ethnically diverse cohort (*n* = 839).

**Results:**

Psychometric properties were adequate. Expected associations with demographics and clinical measures within development and validation cohorts supported validity.

**Conclusions:**

This composite supports memory research across cohorts that administer the UDS and LLTs. A user-friendly UI is freely available to create the score in other datasets.

**Supplementary Information:**

The online version contains supplementary material available at https://doi.org/10.1186/s13195-026-02100-w.

## Background

Alzheimer’s Disease (AD) and Related Dementias (ADRD) research has been accelerated by the standardization of multisite data collection procedures. These datasets include large samples of participants collected across geographic regions, leading to more diverse cohorts, greater analytic power, and enhanced generalizability. In the United States, the National Alzheimer's Coordinating Center (NACC) has led a large-scale effort to standardize data collection across more than 35 Alzheimer’s Disease Centers (ADC) via a Uniform Data Set (UDS) that includes demographic characterization, clinical scales, questionnaires, and neuropsychological assessments [74].

A core element of clinical phenotyping in ADRD is the accurate and robust measurement of memory. The third version of the UDS (UDS v3.0) Neuropsychological Battery was released in 2015 [74] and includes eleven cognitive tasks, of which only the Craft Story and the Benson Figure primarily assess memory. A notable absence from this battery is a list-learning task, one of the most common and effective memory assessments [56]. List-learning tasks can provide a detailed characterization of memory profiles and predict dementia progression [18, 44, 69, 70]. In comparison to other memory paradigms, list-learning tasks have demonstrated equal or superior sensitivity to impairment and have differentiated between cognitive disorders when administered in combination with story memory tasks [2, 44, 57, 75].

ADCs have historically administered a list-learning task, but that task has differed by site. The current version of the UDS (UDS v4.0) Neuropsychological Battery requires sites to choose from one of two list-learning tasks: the Rey Auditory Verbal Learning Test (AVLT) or Consortium to Establish a Registry for Alzheimer's Disease Word List Memory Test (CERAD) [50]. As a result, sites and studies will continue to collect different list-learning tasks. Furthermore, this requirement will introduce differences in list-learning data relative to retrospectively collected UDS v3.0 data.

Although there are practical benefits to allowing ADCs to vary in list-learning task (e.g., comparison to site-specific historic data), a notable downside is limited cross-site comparability. One example of a project affected by such site differences is Diverse Vascular Contributions to Cognitive Impairment and Dementia (DVCID). DVCID is a prospective, observational study of vascular brain injury that is focused on Black/African Americans and Latino/Hispanic Americans. The primary goal of DVCID is to investigate basic mechanisms of small vessel cerebrovascular injury and their relationships with social determinants of health, particularly among individuals of diverse backgrounds [19]. Within DVCID, each study site collects list-learning task data in a manner consistent with their historic procedures, leading to differences.

The study presented in this manuscript, motivated by the desire to develop a harmonized memory metric for the DVCID consortium, sought to leverage best practices in statistical harmonization techniques [6, 29, 39, 47, 66] to create a psychometrically robust memory composite that is on the same scale and interpretable, regardless of the list-learning task administered. To that end, we apply a harmonization and cognitive domain modeling framework described by Mukherjee et al. [47] via an adapted processing script created for a prior harmonization study [29]. This approach relies on linking items, which are test items administered in more than one study. As these items are common among participants from different studies or datasets, they serve as a reference scale for measuring ability. Performance on items that differ across studies or datasets can then be evaluated in relation to the reference scale. Including items that differ across studies is important because those items still inform on the cognitive level for a given person in the study in which the test was administered. The final product combines linking items and study-specific items to produce a single score for each participant that is on the same scale regardless of what tasks they complete. Studies included in this manuscript have the UDS in common and differ in their list-learning tasks. This approach was selected as it creates a reference scale, then separately evaluates how list-learning tasks relate to that scale, before combining all data to create a harmonized score. The separate evaluation of list-learning tasks is necessary as these tasks differ in structure (e.g., delay interval) and difficulty, and it should not be assumed that similar metrics across list-learning tasks measure memory identically.

To address these issues, we pooled data from 8716 participants recruited across four consortia and 19 ADCs to create the UDS Memory plus List-Learning score (UDS-M+). We then tested the performance of this composite in a large independent sample, the DVCID cohort (*n* = 839). We hypothesized that the UDS-M+ will 1) show adequate model fit, reliability, and precision; 2) show evidence of construct validity through associations with other independent cognitive tasks, disease severity, imaging, and plasma phosphorylated tau (ptau) levels. We then converted this processing pipeline into an open-source package that creates the score for datasets not included within this manuscript. The package allows ADCs and other studies to combine list-learning data into a single, harmonized outcome, and does not require a background in harmonization methods.

## Methods

### Participants

As this project was conceived by DVCID, where sites administered one of the five list-learning tasks, the DVCID sample was used as the Validation cohort. To ensure applicability of this score to a range of ADRD diagnoses, we included data from studies of AD (early- and late-onset), frontotemporal dementia (behavioral, language, and motor variants), and cerebrovascular disease. The ADCs associated with DVCID sites were contacted to request list-learning data on other research participants who were not enrolled in DVCID. The final participant pool was recruited from 19 ADCs and four ADRD consortia.

The score was first developed and tested in a diagnostically diverse sample of participants (Developmental cohort; *n* = 8716) recruited through several consortia: ARTFL-LEFFTDS Longitudinal Frontotemporal Lobar Degeneration Study (ALLFTD: NCT04363684), Biomarkers for Vascular Contributions to Cognitive Impairment and Dementia (MarkVCID: NCT06284213), and the Longitudinal Early-Onset Alzheimer's Disease Study (LEADS: NCT03507257). Additional data were also included from ADCs associated with several DVCID sites: University of California, San Francisco, University of California, Davis, 1Florida, University of Wisconsin, Indiana University, and University of Southern California. The UDS-M+ was then tested in a separate cohort of participants enrolled through 16 centers in the DVCID consortium (Validation cohort; DVCID: *n* = 839); these participants did not overlap with those enrolled through the DVCID site ADCs that were included in the Developmental cohort. Participants in both cohorts (Table 1) presented with a range of clinical diagnoses and severity levels (including cognitively unimpaired) that were made in consensus conferences using published criteria [3, 7, 22, 23, 27, 34, 42, 45, 53, 53–55, 58, 59].

**Table 1:** Participant characteristics

	**Overall**	**Developmental**	**DVCID**	
Sample Size	8716	7877	839	
Age (years)	67.68 (11.29)	67.01 (11.53)	73.91 (5.85)*	
Education (years)	15.65 (3.14)	15.63 (3.19)	15.82 (2.57)	
Race/Ethnicity				
White non-Hispanic	5433 (62.6)	5077 (64.8)*	356 (42.5)	
Hispanic or Latino	871 (10.0)	685 (8.7)	186 (22.2)*	
Black/African American	950 (10.9)	660 (8.4)	290 (34.6)*	
Asian	358 (4.1)	357 (4.6)	1 (0.1)	
Other	94 (1.1)	91 (1.2)	3 (0.4)	
Unknown/declined to state	1010 (11.6)	1007 (12.4)	3 (0.3)	
Sex				
Female (%)	4919 (56.4)	4338 (55.1)	581 (69.2)*	
Unknown/declined to state	5 (0.0)	5 (0.1)	0 (0.0)	
CDR-g (%)				
0	3783 (43.4)	3232 (41.0)	551 (65.7)*	
0.5	3255 (37.3)	2968 (37.7)	287 (34.2)	
1	1142 (13.1)	1141 (14.5)	1 (0.1)	
2	325 (3.7)	325 (4.1)	0 (0.0)	
3	24 (0.3)	24 (0.3)	0 (0.0)	
Missing	187 (2.1)	187 (2.4)	0 (0.0)	
CDR-sb	1.97 (2.81)	2.14 (2.90)*	0.39 (0.70)	
Diagnostic Syndrome				
Cognitively Unimpaired	3316 (38.0)	2733 (34.7)	583 (69.5)*	
MCI	1197 (13.7)	996 (12.6)	201 (24.0)*	
AD	1560 (17.9)	1560 (19.8)*	0 (0.0)	
Vascular	95 (1.1)	95 (1.2)	0 (0.0)	
bvFTD	297 (3.4)	297 (3.8)	0 (0.0)	
nfvPPA	43 (0.5)	43 (0.5)	0 (0.0)	
CBS	121 (1.4)	121 (1.5)	0 (0.0)	
PSP	104 (1.2)	104 (1.3)	0 (0.0)	
DLB	113 (1.3)	113 (1.4)	0 (0.0)	
Mixed/Unspecified Dementia	442 (5.1)	433 (5.5)	9 (1.1)	
Psych	59 (0.7)	59 (0.7)	0 (0.0)	
Other	1041 (11.9)	1041 (13.2)	0 (0.0)	
Missing/Does not meet criteria	328 (3.8)	282 (3.6)	46 (5.5)*	
UDS-M+	−0.11 (1.02)	−0.16 (1.04)		0.32 (0.71)*

Presents mean (standard deviation) or count (percent) of demographic variables, diagnoses, and the UDS-M+. Data are presented for the full sample, Developmental cohort, and DVCID

^*^The Developmental and DVCID cohorts are compared via T-tests or*χ*^2^ tests, and the group with significantly larger value is indicated with an asterisk. Comparisons were not completed when cohort sample size was small (n < 10)

The “Mixed/Unspecified Dementia” category included participants with multiple degenerative conditions (e.g., vascular dementia and Alzheimer’s disease) and individuals with dementia due to unknown/unlisted causes. The “Other” category of clinical diagnoses included a range of conditions with low sample sizes including unspecified frontotemporal lobar degeneration, multiple system atrophy, and unspecified primary progressive aphasia. The “Missing/Does not meet criteria” category includes those who were missing diagnoses and those who may have cognitive impairment due to an unknown/unspecified condition

*Abbreviations*: *AD* Alzheimer’s disease dementia syndrome, *bvFTD* behavioral variant frontotemporal dementia, *CDR-sb* Clinical Dementia Rating Scale Sum of Boxes, *CDR-g* Clinical Dementia Rating Scale Global Score, *DLB* Dementia with Lewy bodies, *MCI* Mild cognitive impairment, *nfvPPA* nonfluent-primary progressive aphasia, *PPA* primary progressive aphasia, *CBS* Corticobasal syndrome, *PSP* Progressive supranuclear palsy, *Psych* Suspected psychiatry primary etiology, *UDS-M*+ Uniform Data Set Memory plus List-Learning score, *DVCID* Diverse Vascular Contributions to Cognitive Impairment and Dementia

#### Inclusion/exclusion

All participants in both cohorts completed at least one UDS v3 Neuropsychological Battery memory test and a list-learning task (see Measures section). Inclusion criteria required English as the primary language to remove variance associated with language that may impact task performance. An associated study is underway to evaluate the inclusion of tasks completed in other languages. Written informed consent was obtained from all participants or their legal representative. All studies were approved by the Institutional Review Boards of the consortia or ADC in accordance with institutional guidelines and the Helsinki Declaration.

### Measures

#### List-learning Tasks

 Participants were administered one of five list-learning tasks: the California Verbal Learning Test-II, Standard Form, Form A (CVLT-II) [20], the California Verbal Learning Test-II, Short Form, Form A (CVLT-SF) [20], Hopkins Verbal Learning Test Revised Form 1 (HVLT) [4],CERAD [46],and the AVLT Form A [62]. Due to known differences in forms, data from other list-learning task forms were excluded [15, 28]. All tests began with an immediate recall paradigm in which examiners read a list of words to the participant, who were asked to repeat as many words as possible. This immediate recall paradigm was repeated several times, and the number of words recalled at each trial was recorded. The length of the word list and the number of immediate recall trials varies by task. After a pre-specified delay period, which differs in length by task, participants were asked to freely recall as many words as possible (delayed recall). Participants were then asked to identify correct stimuli in a multiple-choice format (“yes” vs “no”). Responses were labeled as “hits” when participants correctly identified test stimuli and “false positives” when they incorrectly endorsed a foil. As a result, each list-learning task includes the following items: 3–5 separate immediate recall trials, a delayed recall trial, recognition hits, and recognition false positives. Modeling this item-level data are best practice, and is preferred to the creation of a composite score that summarizes items (e.g., sum of list-learning immediate recall) [26].

#### UDS memory measures (linking items)

Participants across all list-learning groups completed the UDS Neuropsychological Battery [74], which includes two memory tasks: a story memory task (Craft Story) with verbatim immediate and delayed recall scores and a visual memory task with a free recall and a recognition trial (correct vs incorrect) of a complex figure (Benson Figure). It also includes the Montreal Cognitive Assessment (MoCA), which provides a brief list-learning memory task with immediate recall, delayed free recall, delayed cued recall, and recognition hits [49]. We also included six orientation items from the MoCA: day, city, date, month, year, and place. Each item is scored as correct or incorrect. Although MoCA orientation items represent a related but separate construct from episodic memory [25], they were included due to their importance for evaluating individuals with more severe memory-related impairments [37, 76, 77]. Together, these three tests provide six memory items and six orientation items that are common across all contributing data sources. Two of the six orientation items (day, city) were removed due to poor fit or weak/negative loadings within models. Further investigation of these items suggested that a lack of variance in responses may be driving the poor fit (see Model Building and Supplementary materials).

#### Additional cognitive tasks

Participants completed additional UDS measures: Verbal Fluency, Trail Making Test A, Trail Making Test B, Number Span Forward, Number Span Backwards, and the Multilingual Naming Test (MINT) [5, 74]. Tasks of executive functioning were summarized into a single executive composite score, the UDS3-EF [64], which was created using similar item response theory methods to those used to generate the UDS-M+. A subset of participants (*n* = 227) completed an independent associative memory task, the Tablet-based Cognitive Assessment Tool (TabCAT) Favorites test [68]. The outcome for TabCAT Favorites was total correct responses across immediate and delayed recall conditions. Another subset of participants (*n* = 982) completed an additional story memory test: Logical Memory A of the Wechsler Memory Test-Revised [73], which includes immediate and delayed recall scores.

#### Clinical dementia rating scale (CDR)

Participants’ clinical impairment was rated using the CDR, a clinician-administered, semi-structured interview with the participant and a study partner [46]. Clinicians query the following six areas and provide a rating (values: 0, 0.5, 1, 2, 3): memory, judgment and problem solving, community affairs, home and hobbies, orientation, and personal care. The CDR is commonly used in ADRD research and within AD clinical trials. A weighted algorithm combines domain scores into a global score (CDR-g, range: 0, 0.5, 1, 2, 3) and sum of boxes (CDR-sb; range: 0–18), with higher scores indicating greater impairment.

#### Plasma biomarkers

A subset of Developmental cohort participants (*n* = 29) provided blood samples at the University of California, San Francisco. Plasma ptau 217 (ptau-217) was measured with electrochemiluminescence-based assays on the Meso Scale Discovery platform (MSD, Rockville, MD, USA) using previously published methods [60].

A subset of DVCID participants (*n* = 425) provided blood samples that were processed through DVCID, and the Quanterix Single Molecule Array (SiMoA) assay was used to quantify plasma ptau 181 (ptau-181). These methods are fully described elsewhere [19].

#### Imaging

Positron emission tomography (PET) data were binarized as normal or elevated by the NACC; see https://naccdata.org/data-collection/forms-documentation/biomarker-imaging for additional information [5, 10, 72]. There were 545 participants with amyloid PET results and 71 participants with tau PET results available for this manuscript. All participants with PET data were in the Developmental cohort.

A sample of participants within the Developmental cohort (MarkVCID; *n* = 446) and Validation cohort (DVCID; *n* = 736) underwent brain MRI with the same acquisition protocol [19]. MRI acquisition and processing are fully described online (https://markvcid.partners.org/markvcid1-protocols-resources) and in prior publications [43]. Imaging correlates of the memory composite were assessed using a priori regions of interest in subsamples with available processed volumetric MRI data. Given the central role of the medial temporal lobe in supporting episodic memory, we created a medial temporal lobe (MTL) ROI comprising bilateral hippocampal, entorhinal, and parahippocampal regions [1, 21, 24]. An occipital lobe ROI was created as a control region [8].

### Confirmatory factor analysis with item banking approach for statistical co-calibration

#### Preprocessing

This study followed the framework described by Mukherjee et al. [47]. Preprocessing steps included identification of memory variables and linking items, as well as confirmation that the same forms were used across sites. Data quality control checks included alignment of scoring criteria, demographics, and other variables common across data sources. Data were restricted to the first timepoint at which participants completed a list-learning task and had the opportunity to complete at least one of the UDS Neuropsychological Battery memory items. In the small subset of participants who were missing valid list-learning data, we included the first timepoint they completed UDS measures. Prior to model creation, continuous variables were recoded into ordinal scores with up to 10 response categories and at least 5 observations in each category to facilitate use of a graded response model approach [61]. The equal interval approach we used to recode variables retains the shape of the distribution of the continuous data.

#### Model building

An item-banking confirmatory factor analysis (CFA) approach to harmonization was conducted based on Mukherjee et al. [47] and other prior publications [29, 71]. In this study, we divided the sample into five groups based on which list-learning task participants completed: CVLT-II, CVLT-SF, CERAD, HVLT, AVLT. All groups were also administered the linking items from the UDS Neuropsychological Battery. Model building involved the following steps, repeated for each list-learning group.

We fit a series of bifactor models within each of the five list-learning groups. The models initially attempted to include all list-learning task trials and all linking items. Bifactor models were selected to account for secondary data structures. The first bifactor model included three sets of secondary or methods factors: one for the list-learning items, another for the Craft Story variables, and a third for the MoCA variables. We also considered splitting the MoCA secondary factor into one secondary factor for the orientation items and another for the word-list items. Loadings onto the overall memory factor and the secondary factors were removed if they were negative or led to model convergence issues. The magnitude and significance of loadings was also considered. We followed these same steps when fitting models based in all subsamples (e.g., CVLT-II subsample). Each list-learning task model was evaluated separately. To determine model fit, we used a Weighted Least Squares with Mean and Variance (WLSMV) estimator [47].

For the current study, the initial model (i.e., anchor model) was fit to the AVLT subsample. The AVLT was also used in the anchor model of other harmonization studies [47] and is one of the two memory tests allowed in UDS v4.0. Furthermore, the AVLT was notable for including a range of memory impairment, from cognitively unimpaired individuals to those likely to have significant memory difficulties (e.g., AD, high CDR-g). Prior research has shown that varying the order of models in a co-calibration harmonization study does not meaningfully affect the quality of factor scores produced through an item-banking CFA [29]. However, we repeated all modeling steps using the CERAD as the initial model to confirm that varying the model order did not meaningfully affect results (see Supplementary materials).

Following this initial model, we separately determined the factor structure of each list-learning subsample. We then began linking models. The parameter estimates for the anchor model (AVLT variables) were recorded, and estimates for all linking items were retained in subsequent models. A subsequent model was then fit to another subset of data (i.e., a new list-learning task), in which the thresholds and loadings for the common linking items were fixed to their corresponding values from the AVLT model. Thresholds and loadings for variables from the second list-learning task were freely estimated. This procedure was repeated for all list-learning subsamples until parameters were estimated for all list-learning variables, based on their previously defined factor structure (see above). All final list-learning models included secondary factors. These models only used data from the Developmental cohort. This final model, in which all parameters were fixed and included the entire Developmental cohort, then calculated a single memory factor score that is on the same scale for each participant regardless of which list-learning task was administered. Factor score creation utilized a Maximum Likelihood Robust (MLR) estimator and theta parametrization.

#### Model fit

The fit of each model was evaluated based on thresholds used in prior harmonization studies: the Tucker-Lewis Index (TLI; adequate: > 0.90; good: > 0.95), the Comparative Fit Index (CFI; adequate: > 0.90; good: > 0.95), and the Root Mean Square Error of Approximation (RMSEA; adequate: < 0.08; good: < 0.05) [32, 47]. Model building was completed in Mplus [38] via Stata Statistical software [65] using the RUNMPLUS Stata package [36]. All other statistical analyses (e.g., regressions, descriptives) were conducted in R (v4.2.3) [67].

### Statistical analyses

To test for differences between the Developmental and Validation cohorts on key participant characteristics, t-tests or *χ*^2^ tests compared cohorts on demographic variables, clinical impairment, diagnoses, and UDS-M+ scores.

#### Model precision

Measurement precision of factor scores can vary across ability levels and its precision can be quantified through a metric, item information, which can be summed across all items to create a test information statistic. We provide test information plots [16] for the full sample, then delineated by list-learning group.

#### Validity analyses

To evaluate evidence for convergent construct validity, separate linear regressions were fit to test the association of the UDS-M+ and each of the following: age, sex, education, CDR-sb, and independent memory measures (TabCAT Favorites, Logical Memory). Each regression adjusted for the CDR-sb, unless CDR-sb was the primary variable of interest. Linear regressions were fit to examine whether disease severity (CDR-g) was associated with UDS-M+ scores. CDR-g was entered as a categorical variable and separate regression analyses evaluated pairwise comparisons: 0 vs 0.5; 0.5 vs 1 +; 0 vs 1 +. Post-hoc linear regressions investigated the relationship between the UDS-M+ and the CDR Memory domain box score. Discriminant construct validity was evaluated by testing associations with non-memory cognitive tasks [64]. Analyses were first completed in the Developmental cohort then replicated as possible within the Validation cohort.

Given that memory deficits are an early and common consequence of AD pathology, the association between the UDS-M+ score and log(10)-transformed plasma ptau levels was tested via regressions that controlled for age, sex, and education. In this analysis, the Developmental cohort was limited to those with CDR-g of 0 and 0.5 to allow for a more accurate comparison to the Validation cohort which had a restricted range of disease severity (CDR-g < 1).

Linear regressions compared UDS-M+ scores across amyloid PET positivity (binarized) and tau PET positivity (binarized), controlling for age, sex, and education. Post hoc analyses repeated these comparisons within a second subsample that was restricted to those diagnosed as CU, MCI, or AD.

The relationship between UDS-M+ and imaging (MTL, occipital) was examined using linear regressions controlling for age, total intracranial volume, education, and CDR-sb.

#### Supplementary analyses

This project was completed with a combination of three statistical programs, two of which are proprietary. The script that creates the memory score is also complicated, requiring knowledge of several coding languages as well as a background in item response theory and harmonization methods. To facilitate usability, all code was converted to R, a free software, and memory scores were calculated via the MIRT package [11]. All parameters (e.g., loadings, thresholds) from the Mplus model were converted into a useable format for MIRT. The resulting MIRT-based memory score was correlated with the original, Mplus-based score within the Developmental cohort. We then repeated all analyses within this manuscript using the MIRT-based memory score. We provide a summary of these analyses in the Supplementary materials. The MIRT-based script has been converted into a user-friendly UI via the Rshiny package [12]. This package requires the user to select a dataset and identify variables via a dropdown menu. The user does not need a background in any coding language, item response theory, or harmonization methods.

## Results

### Sample characteristics

Participant characteristics are provided in Table 1. Compared with the Developmental cohort, DVCID participants were significantly older, and less impaired on the CDR-sb. Sample characteristics delineated by list-learning task are presented in Supplementary Table 1.

### Model building and fit

In the initial model (AVLT), the standardized loadings between linking items and the overall memory factor ranged from 0.25 to 0.86. Linking item loadings were carried forward to all other list-learning models. All models demonstrated adequate or excellent fit statistics (CFIs > 0.97; TLIs > 0.98; RMSEA range: 0.02 to 0.052). Fit statistics are summarized in Supplementary Table 2.

### Model precision

**Fig. 1:**
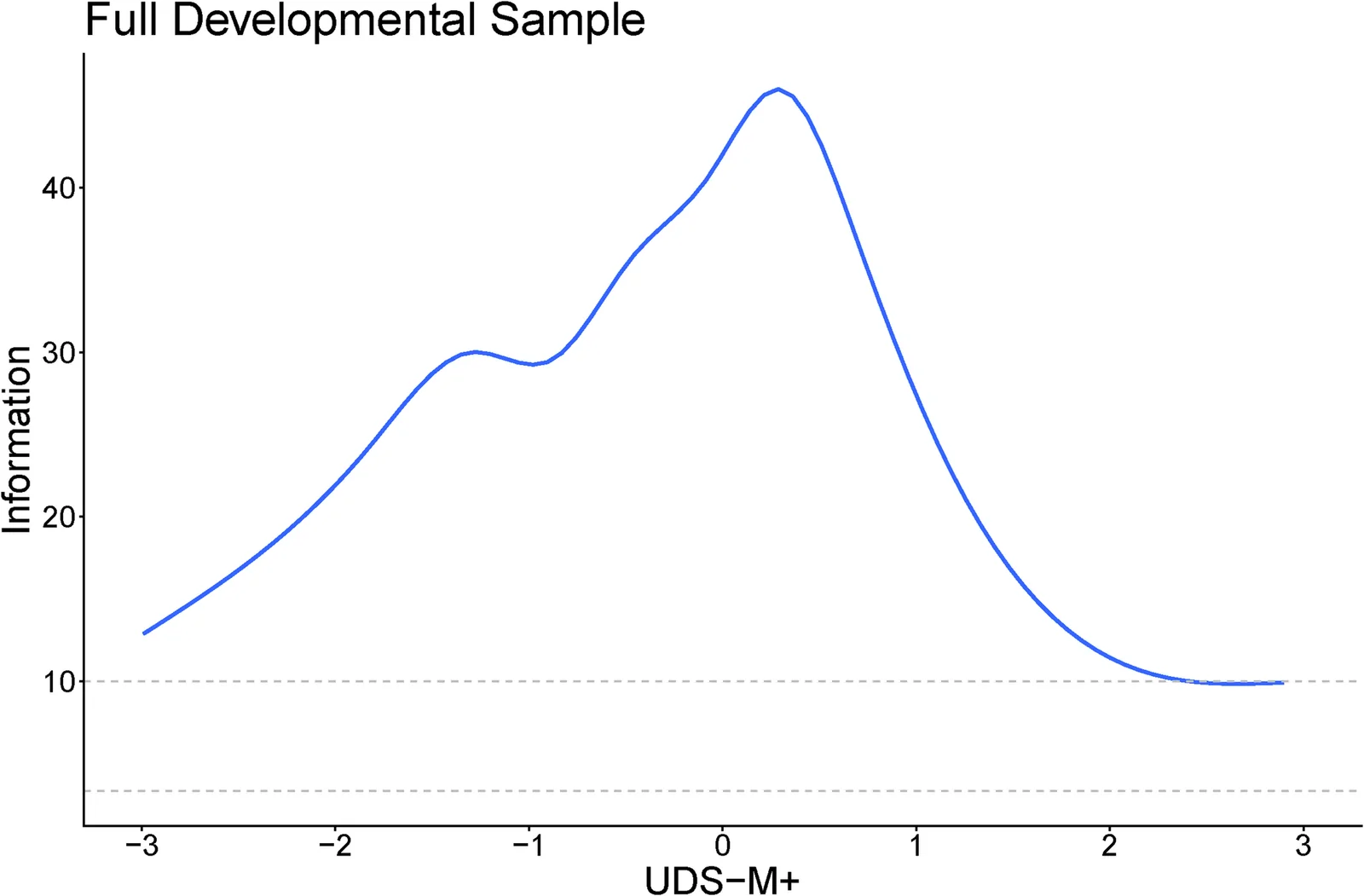
Model precision within the Developmental cohort. Presents a test information plot for the UDS-M+ derived from the Developmental cohort. Gray dashed lines are placed at information = 10 (corresponding to a reliability of 90%) and 3.33 (corresponding to a reliability of 70%)

The test information plot (Fig. 1) shows that approximately 92% of the sample has information greater than 10 (corresponding to a reliability of 90%), and 99% of the sample has information greater than 3.33 (corresponding to a reliability of 70%) [51]. A test information plot delineated by list-learning task group is presented in Supplementary Fig. 1.

### Associations with clinical measures

**Fig. 2:**
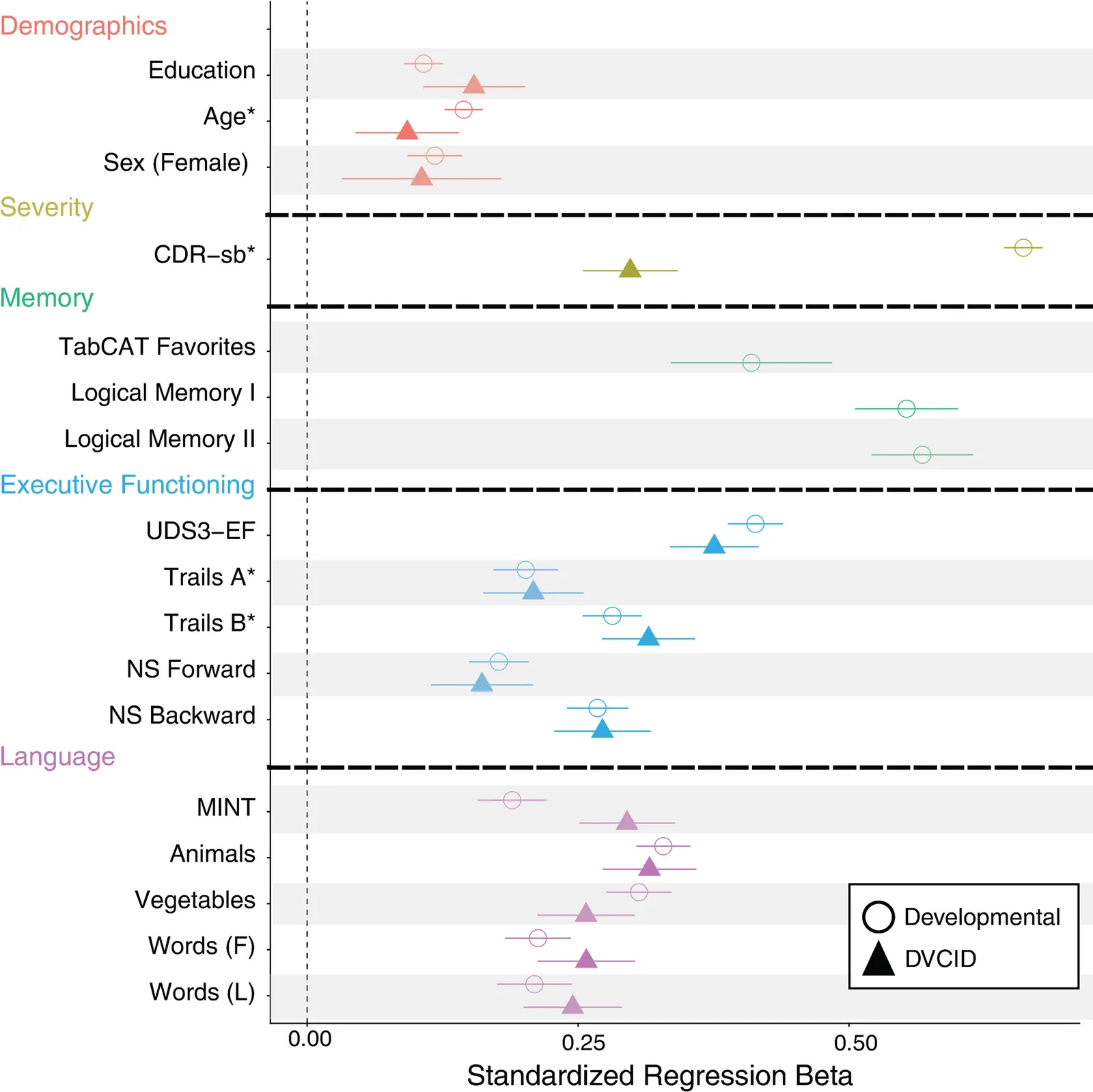
Convergent and discriminant validity. Forest plot presenting standardized regression betas in the Developmental (circle) and DVCID cohorts (triangle), after controlling for CDR-sb, with the exception of the CDR-sb row. Confidence intervals (95%) are shown via the horizontal lines. Variables are color coded by domain. * indicates reverse scoring. Abbreviations: CDR-sb: Clinical Dementia Rating scale sum of boxes; NS: Number Span;; MINT: Multilingual Naming Test; TabCAT: Tablet-based Cognitive Assessment Tool; Trails A: Trail Making Test A; Trails B: Trail Making Test B; NS: Number Span; UDS-EF: Uniform Data Set (v3.0) executive function composite score; Words: Verbal Fluency; DVCID: Diverse Vascular Contributions to Cognitive Impairment and Dementia

**Fig. 3:**
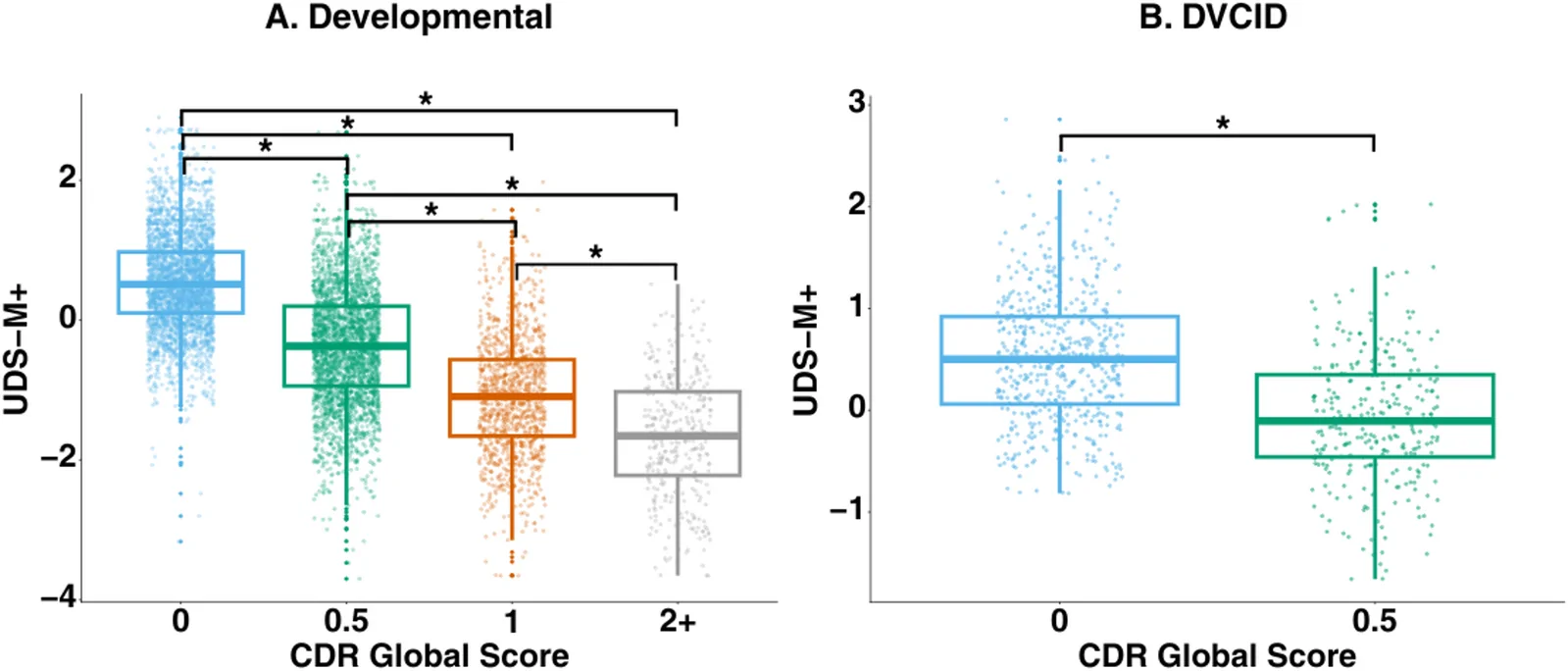
UDS-M+ by Clinical Dementia Rating Scale These graphs illustrate the stepwise decline in UDS-M+ scores that was observed with increasing disease stage (*p* < 0.001) in two independent cohorts. Abbreviations: CDR-g: Clinical Dementia Rating Scale global score; DVCID: Diverse Vascular Contributions to Cognitive Impairment and Dementia; UDS-M+ : Uniform Data Set Version 3 Memory plus List-Learning score

Associations between the UDS-M+ and demographics and clinical measures are displayed as standardized regression coefficients in Fig. 2. Within the Developmental cohort, higher UDS-M+ scores were associated as expected with younger age (β = −0.14; 95% CI: −0.13, −0.16; *p* < 0.001), greater years of education (β = 0.11; 95% CI: 0.09, 0.13; *p* < 0.001), and female sex (β = 0.12; 95% CI: 0.09, 0.14; *p* < 0.001). Lower scores were associated with greater clinical impairment, as measured by the CDR-sb (β = −0.66; 95% CI: −0.64, −0.68; *p* < 0.001). The UDS-M+ was strongly correlated with all independent memory tests (TabCAT Favorites: β range: 0.42 to 0.58; *p* < 0.001), whereas the magnitude of associations with measures of executive functioning (β range: 0.17 to 0.41) and language (β range: 0.19 to 0.32) were relatively lower. There was a stepwise decline (Fig. 3) in UDS-M+ performance across CDR-g scores (CDR 0 > 0.5 > 1 > 2 +; p’s < 0.001) and Memory domain box scores (0 > 1 > 2 +; *p* < 0.001). As shown in Figs. 2 and 3, the UDS-M+ associations showed the same general pattern of results in the Developmental and Validation cohorts. One association was notably different between the cohorts. The association between the UDS-M+ and CDR-sb in the Developmental cohort was larger than the association in the DVCID cohort (r = −0.66; 95% CI: −0.64, −0.68 vs r = −0.30; 95% CI: −0.25, −0.34). As noted in Table 1, the cohorts differ in the average and distribution of CDR-sb, with DVCID representing a less impaired or less symptomatic group with restricted variance.

### Associations with ptau, PET positivity, brain volume

**Fig. 4:**
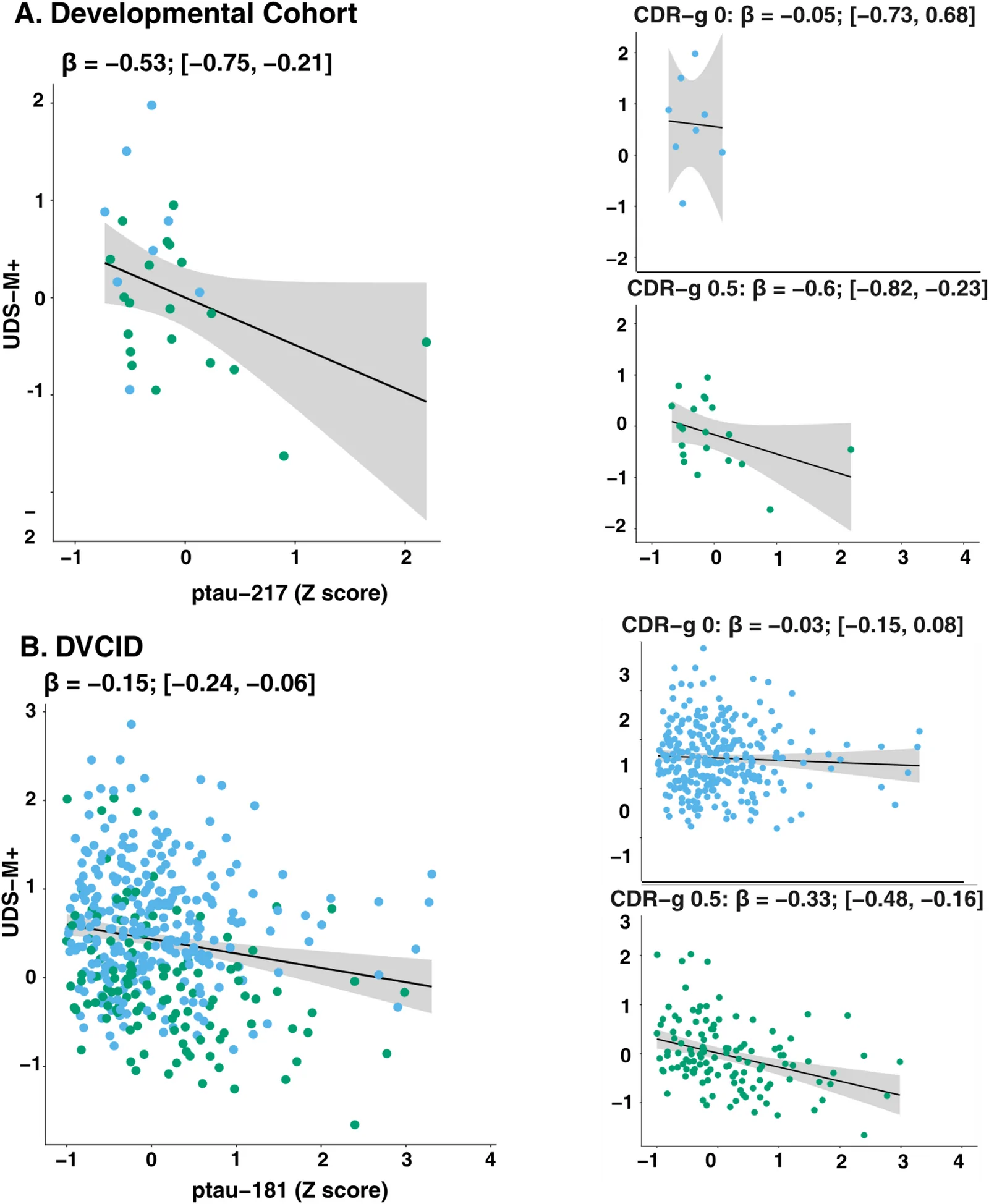
Association between UDS-M+ and plasma phosphorylated tau levels. Each graph presents the association between UDS-M+ and plasma phosphorylated tau levels (Developmental cohort: ptau-217; DVCID: ptau-181), after controlling for age, sex, education, and CDR-sb. Graphs on the left present the results for the Developmental cohort (**A**) and (**B**) DVCID cohort. The stacked graphs on the right display the same results, separated by CDR-g. Ptau levels were log transformed then Z scored. Abbreviations: ptau: phosphorylated tau; CDR-g: Clinical Dementia Rating scale global score; CDR-sb: Clinical Dementia Rating scale sum of boxes; DVCID: Diverse Vascular Contributions to Cognitive Impairment and Dementia; UDS-M+ : Uniform Data Set Memory plus List-Learning score

Worse performance on the UDS-M+ was strongly associated with higher plasma ptau-217 levels in the Developmental cohort (Fig. 4A), after controlling for age, education, sex, and CDR-sb (*β* = −0.53; 95% CI: −0.75, −0.21; *p* < 0.001). Similarly, in DVCID (Fig. 4B), worse performance on the UDS-M+ was associated with higher plasma levels of ptau-181 (β = −0.15; 95% CI: −0.24, −0.06; *p* = 0.002), with the strongest association in the CDR-g = 0.5 group (β = −0.33; 95% CI: −0.47, −0.16; *p* < 0.001).

**Fig. 5:**
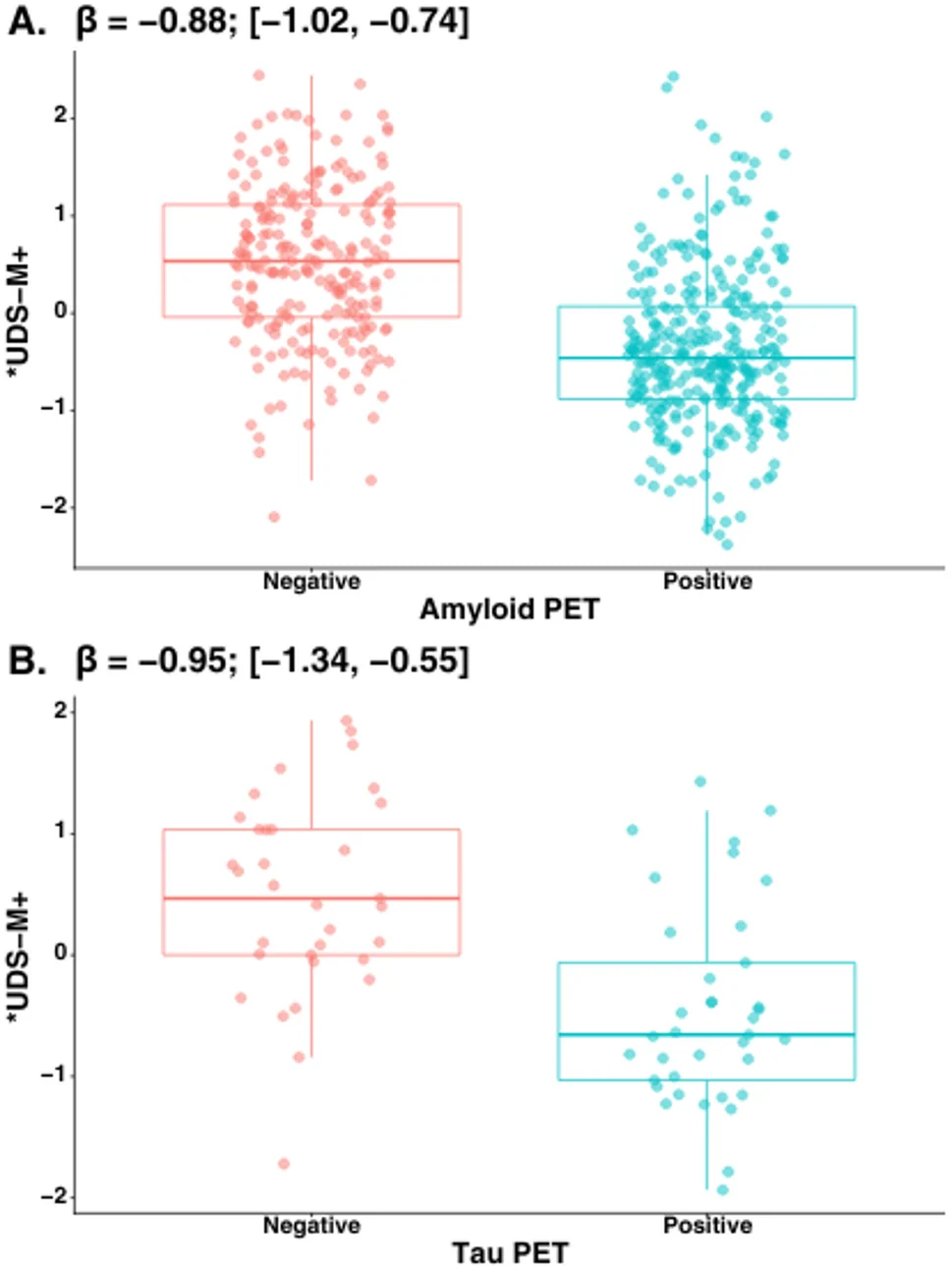
Association of UDS-M+ with amyloid PET and tau PET positivity. Boxplots present UDS-M+ scores for **A** amyloid PET positive (red) vs negative (blue) participants as well as **B** tau PET positive (red) and negative (blue) participants. *UDS-M+ scores have been residualized for age, sex, and education. Standardized beta values [95% confidence intervals] are presented at the top of each figure. Data are from the Developmental cohort. Abbreviations: UDS-M+ : Uniform Data Set Memory plus List-Learning score; PET: positron emission tomography

The UDS-M+ significantly differed between PET positive and negative groups in the expected directions (Fig. 5). Amyloid PET positive participants had significantly lower UDS-M+ scores than amyloid negative participants (Fig. 5A: *β* = −0.88; 95% CI: −1.02, −0.74; *p* < 0.001). Tau positive participants also scored significantly lower than tau negative participants (Fig. 5B: *β* = −0.95; 95% CI: −1.34, −0.55; *p* < 0.001). Results remained similar in a post hoc analysis restricting the sample to those diagnosed as CU, MCI, or AD (amyloid: *β* = −1.04; 95% CI: −1.22, −0.87; *p* < 0.001; tau: *β* = −1.12; 95% CI: −1.54, −0.69; *p* < 0.001). These analyses were only completed in the Developmental cohort.

Lower UDS-M+ scores were associated with smaller MTL volumes in the Developmental cohort (β = 0.20; 95% CI: 0.11, 0.30; *p* < 0.001) and DVCID (β = 0.10; 95% CI: 0.04, 0.16; *p* = 0.001), among participants without significant disease severity (CDR-g < 1). Supporting divergent validity, the UDS-M+ was not significantly associated with the occipital region (β = 0.01; 95% CI: −0.06, 0.07; *p* = 0.78).

### Supplementary results

In sensitivity analyses, the CERAD model was used to estimate parameters instead of the AVLT model. The resultant factor score (CERAD-first) was correlated at 0.99 (Supplementary Fig. 2) with the primary factor score (AVLT-first) that is presented in the results section of this manuscript. Results are fully described in the Supplementary materials and presented within Supplementary Tables 3–4. Additional supplementary analyses demonstrating distribution of UDS-M+ scores across CDR groups are presented in Supplementary Fig. 3.

The MIRT-based memory score was strongly correlated with the Mplus-based memory score (Supplementary Fig. 4; r = 0.99; 95% CI: 0.99, 0.99; *p* < 0.001). In a sensitivity analysis, all analyses were reconducted using the MIRT-based memory score. The same pattern of findings was observed, with very similar parameter estimates (Supplementary Table 5).

## Discussion

We present the development and validation of the UDS-M+, a memory composite score developed using modern psychometric methods [16, 29, 48]. The UDS-M+ was built in a diagnostically heterogeneous sample to support generalizability, and the resulting score showed good model fit with moderate-to-strong factor loadings. Model fit and loadings were within the same range as other, gold-standard harmonization studies [13, 15, 26, 47]. Construct validity was supported by strong associations in the hypothesized directions with demographic factors (e.g., age), independent memory tasks, MTL volume, and plasma ptau levels. Scores differed as expected across groups defined by clinical impairment (CDR-g) and biomarkers (amyloid and tau PET positivity). The robustness of these findings is bolstered by replication within an independent validation cohort, DVCID.

An important feature of the UDS-M+ is that the resulting memory score is on the same scale regardless of which list-learning task was administered. This feature reflects a significant improvement over the use of raw scores or standardized scores, which are not directly comparable across tests or cohorts [31, 35, 40]. For example, a CVLT-II delayed recall of nine words does not equate to a CVLT-SF delayed recall of nine words, as there are notable differences in task demands (e.g., word-list length, recall interval, stimuli). An alternative harmonization approach is to convert scores on each list-learning task to Z scores based on the distribution of the list-learning task within each sample. A complication with Z score comparisons is that the list-learning tasks likely differ in distribution and, like raw scores, Z scores do not account for differences in task demands. For example, if participant A (CVLT-II) and participant B (CVLT-SF) both have a Z score of 0, it does not necessarily mean that they have equal memory ability, just that they have average scores within their respective subsamples. The UDS-M+, in contrast, was created with item response theory methods that allow for differences in task demands and place each participant’s score on the same scale. As a result, if participant A (CVLT-II) had a UDS-M+ score of 0 and participant B (CVLT-SF) had a UDS-M+ score of 0, we can conclude that participant A’s memory is measured to be equal to that of participant B (with a margin of error).

Test information plots suggest the UDS-M+ is a reliable measure across the range of memory ability. The factor score performs particularly well among sample participants who are at average or below average memory ability. There is a decline in precision at the right end of the UDS-M+ distribution, although item information was still generally acceptable. This decrease in precision may be due to potential ceiling effects in some tasks. When individuals score perfectly on some measures, it prevents that task from differentiating among higher scoring individuals, leading to decreased model precision. Ceiling effects are a known issue in cognitive testing [30, 63] and likely contribute to study-specific choices in list-learning tasks. Studies evaluating more impaired participants may select easier list-learning tasks and those evaluating higher performing samples may select more difficult tasks. The UDS-M+ allows for combining participants from these disparate studies as all participants’ memory abilities are on the same scale. Moreover, if a study switches list-learning tasks as a participant declines, for example from the CVLT-II to the CVLT-SF, researchers will still be able to compare to historic data. The ability to evaluate memory performance regardless of list-learning task will become particularly important as the UDS v4.0 requires investigators to administer either the AVLT or CERAD [50]. As a result, many UDS sites who have historically administered a different list-learning task will face a dilemma: they may switch to a new task or administer two list-learning tasks. Switching to a new task disrupts alignment with all retrospectively collected data, while administering two tasks is burdensome for participants and staff and potentially introduces interference effects. This memory composite may offer a solution, as the UDS-M+ can be used to harmonize the AVLT and CERAD with other tasks. Of note, another harmonization effort has included these two list-learning tasks and is another potential solution [47, 63].

This project adds to several ongoing studies [14, 17, 41] that have applied statistical harmonization approaches to enable the inclusion of non-overlapping cognitive tests across sites and projects. For example, the item-banking code used in this study was adapted from an effort to harmonize cognitive instruments administered in six countries through the Health and Retirement Study’s Harmonized Cognitive Assessment Protocol (HRS-HCAP) and has been applied to other longitudinal cohorts [29, 39, 47, 63, 71, 78]. A framework for cognitive harmonization was provided by Mukherjee et al., [47] and its companion article Mukherjee et al. (2020) [48]. The current study followed this protocol to address the goal of harmonizing list-learning task data, and builds upon prior work by producing a user-friendly application for interested investigators. The need for cognitive harmonization, and data harmonization more broadly, continues to grow in importance as the field moves increasingly towards multi-center and multi-consortia studies [9, 33, 64], as harmonization enables cross-national research and examination of more complex questions.

We present within the Supplementary materials a second version of the UDS-M+ (MIRT-based). Although the Mplus-based and MIRT-based versions are not interchangeable, they result in identical patterns of results, demonstrating reliability and validity. The goal in creating this second version of the UDS-M+ was to do so in a reproducible way that allows others to create a score even if they lack a background in harmonization or coding. To that end, we have developed a UI via the Rshiny package where users can select a dataset and identify variables via a dropdown menu. The program then cleans the data, provides descriptives, highlights potential issues (e.g., high degree of missingness), creates the memory score, and returns that score to the uploaded dataset. As a result, ADCs and individual studies can independently generate this score for any sample in real time, without requiring expertise in harmonization methods or waiting on input from an external group. This Rshiny program is accessible on Github (https://github.com/Cognitive-Composites). 

There are limitations to the current version of this score. Precision was sufficient across all list-learning task cohorts, though estimates were lower at the extremes of latent memory ability. We primarily present cross-sectional data and have not systematically investigated longitudinal change on the UDS-M+. Ongoing longitudinal analyses will be presented in a separate publication and will include estimated rates of meaningful change in UDS-M+ across controls and diagnostic groups. Additionally, some cross-sectional analyses are limited as the samples with available biomarker data (e.g., plasma p-Tau, Tau PET) were modest in size. Although results from the Developmental cohort support the use of the score across a range of disease severities and diagnoses, and replication in DVCID support its use with participants identifying as Black/African American or Latino/Hispanic American, additional studies are required to extend the UDS-M+ for use in other testing languages and cultures. Work is underway to develop a version of the score for Spanish speakers. As there may be meaningful differences in how cultures complete linking items, this language-focused study will include measurement invariance testing and specific Differential Item Functioning analyses to evaluate linking items across cohorts. Until these analyses are completed, generalizability of the UDS-M+ to non-English-speaking and/or non-US based populations is limited. The UDS-M+ was created to aid in comparing memory scores across participants but should not be interpreted as a normatively corrected estimate of memory ability. A score of 0, for example, is relative to this mixed diagnostic group, and should therefore not be interpreted as representing average memory ability at a population level. We also do not provide demographic adjustments, and recommend this step be conducted by investigators in their statistical models based on the relevant confounds in their study. Although the score can accommodate five of the most common list-learning tasks, there are other measures that were not included. The code used to generate the UDS-M+, however, is designed to be iterative, allowing for the addition of other list-learning tasks and memory measures in the future. 

## Conclusion

We have successfully applied item-banking methods to harmonize list-learning task data across several consortia and ADCs. The result of this project is a factor score that is on the same scale regardless of which list-learning task was administered, which will be useful for cross-center and cross-consortium ADRD research. To further increase usability, we have created a user-friendly UI that allows investigators to generate this score independently and in real time, via dropdown menus and without any background in coding or harmonization. Future work will develop this score for use in other languages.

## Supplementary Information

Supplementary Material 1.

## Data Availability

Much of the data analyzed during the current study is recorded with the National Alzheimer’s Coordinating Center [https://www.naccdata.org/]. Additional data are available through the following consortia: ALLFTD ([https://www.allftd.org/]), LEADS ([https://leads-study.medicine.iu.edu/]), MarkVCID ([https://markvcid.partners.org/acknowledgements]), and DVCID ([https://diversevcid.ucdavis.edu/]) consortia. Study-specific data from UCSF is obtainable through standardized procedures ([https://data.ucsf.edu/data-sharing]). An Rshiny script is available on Github to create the UDS-M+ (https://github.com/Cognitive-Composites).

## Abbreviations

AD: Alzheimer’s Disease
ADRD: Alzheimer’s Disease and Related Dementias
ADC: Alzheimer’s Disease Centers
NACC: National Alzheimer’s Coordinating Center
UDS: Uniform Data Set
AVLT: Rey Auditory Verbal Learning Test
CERAD: Consortium to Establish a Registry for Alzheimer’s Disease Word List Memory Test
DVCID: Diverse Vascular Contributions to Cognitive Impairment and Dementia
UDS-M+: UDS Memory plus List-Learning score
Ptau: Plasma phosphorylated tau
ALLFTD: ARTFL-LEFFTDS Longitudinal Frontotemporal Lobar Degeneration Study
LEADS: Longitudinal Early-Onset Alzheimer's Disease Study
CVLT-II: California Verbal Learning Test-II, Standard Form
CVLT-SF: California Verbal Learning Test-II, Short Form
HVLT: Hopkins Verbal Learning Test Revised
MoCA: Montreal Cognitive Assessment
MINT: Multilingual Naming Test
TabCAT: Tablet-based Cognitive Assessment Tool
CDR: Clinical Dementia Rating Scale
CDR-g: Clinical Dementia Rating Scale global score
CDR-sb: Clinical Dementia Rating Scale sum of boxes
PET: Positron emission tomography
MTL: Medial Temporal Lobe
ROI: Region of Interest
CFA: Confirmatory Factor Analysis
MLR: Maximum Likelihood Robust Estimator
TLI: Tucker-Lewis Index
CFI: Comparative Fit Index
RMSEA: Root Mean Square Error of Approximation
