# Supplementary material for: Development and validation of a harmonized memory score for multicenter Alzheimer’s disease and related dementia research
Source: Alzheimers Res Ther. 2026 Jun 15;18:209. doi: 10.1186/s13195-026-02100-w (PMC13595592; doi:10.1186/s13195-026-02100-w)
Supplement: Supplementary file 1 [file 13195_2026_2100_MOESM1_ESM.docx]

| **Table of Contents** | |
| --- | --- |
| **Model building** | **Page 2** |
| **CERAD as Anchor Model** | **Page 3** |
| **Supplementary Tables** | **Page 4** |
| **Supplementary Figures** | **Page 18** |

1. **MIRT-based memory score**

To facilitate use of this score, we created a MIRT-based (Chalmers 2012) metric within a publicly available software based on the loadings and thresholds from Mplus. This score is accessible through a user-friendly UI developed through the RShiny package (Chang et al. 2012) and is available on Github (<https://github.com/Cognitive-Composites>). To validate this score, we first correlated it with the original UDS-M+ (Supplementary Figure 5). We then repeated all manuscript analyses with this MIRT-based memory score; a selection of the results is presented in Supplementary Table 5. For additional comparisons, please contact the authors.

2. **CERAD as Anchor Model**

We completed a sensitivity analysis in which we used the CERAD list-learning task instead of the AVLT list-learning task as the anchor model (Model 1) whose linking item parameters were carried forward into all subsequent models. All models used item-level list-learning data and otherwise were identical to the models which used the AVLT in Model 1. We then correlated the resulting factor score from this CERAD-first model with the factor score created by the AVLT-first model. We also compared thresholds and loadings between models. Despite some differences in individual loadings and thresholds, the resultant factor scores were correlated at 0.99. Loadings and thresholds for the CERAD-first and AVLT-first models are presented below (Supplementary Tables 3-4).

3. **Supplementary Tables**

| Supplementary Table 1: Sample demographics for the combined Developmental and DVCID cohorts | | | | |  |  |
| --- | --- | --- | --- | --- | --- | --- |
|  | AVLT | CVLT-II | HVLT | | CVLT-SF | CERAD |
| Sample Size | 1885 | 1444 | 944 | | 2883 | 1230 |
| Age (years) | 63.12 (10.28) | 67.49 (10.50) | 72.32 (7.14) | | 65.82 (12.04) | 75.76 (7.48) |
| Education (years) | 15.91 (2.51) | 17.00 (2.33) | 15.55 (2.82) | | 15.67 (2.90) | 13.99 (4.33) |
| Race/Ethnicity |  |  |  | |  |  |
| White non-Hispanic | 835 (44.3) | 1165 (80.7) | 399 (42.3) | | 2302 (79.8) | 581 (47.2) |
| Hispanic or Latino | 44 (2.3) | 19 (1.3) | 405 (42.9) | | 0 (0.0) | 299 (24.3) |
| Black/African American | 393 (20.8) | 65 (4.5) | 124 (13.1) | | 63 (2.2) | 292 (23.7) |
| Asian | 8 (0.4) | 85 (5.9) | 4 (0.4) | | 224 (7.8) | 36 (2.9) |
| Other | 2 (0.1) | 18 (1.2) | 6 (0.6) | | 56 (1.9) | 12 (1.0) |
| Unknown/declined to state | 603 (31.9) | 92 (6.4) | 6 (0.6) | | 231 (8.2) | 10 (0.8) |
| Sex |  |  |  | |  |  |
| Female (%) | 1173 (62.2) | 782 (54.2) | 593 (62.8) | | 1421 (49.3) | 752 (61.1) |
| CDR-g (%) |  |  |  | |  |  |
| 0 | 1167 (61.9) | 998 (69.1) | 455 (48.2) | | 458 (15.9) | 515 (41.9) |
| 0.5 | 534 (28.3) | 408 (28.3) | 438 (46.4) | | 1307 (45.3) | 447 (36.3) |
| 1 | 114 (6.0) | 35 (2.4) | 46 (4.9) | | 837 (29.0) | 99 (8.0) |
| 2 | 11 (0.6) | 3 (0.2) | 5 (0.5) | | 262 (9.1) | 40 (3.3) |
| 3 | 1 (0.1) | 0 (0.0) | 0 (0.0) | | 19 (0.7) | 3 (0.2) |
| Missing | 58 (3.1) | 0 (0.0) | 0 (0.0) | | 0 (0.0) | 126 (10.2) |
| CDR-sb | 0.95 (1.82) | 0.56 (1.21) | 0.98 (1.55) | | 3.86 (3.27) | 1.66 (2.64) |
| Diagnostic Syndrome |  |  |  | |  |  |
| Cognitively Unimpaired | 1258 (66.7) | 834 (57.8) | 457 (48.4) | | 230 (8.0) | 355 (28.9) |
| MCI | 207 (11.0) | 267 (18.5) | 201 (21.3) | | 420 (14.6) | 16 (1.3) |
| AD | 262 (13.9) | 29 (2.0) | 109 (11.5) | | 839 (29.1) | 291 (23.7) |
| Vascular | 0 (0.0) | 1 (0.1) | 18 (1.9) | | 31 (1.1) | 35 (2.8) |
| bvFTD | 8 (0.4) | 7 (0.5) | 3 (0.3) | | 271 (9.4) | 4 (0.3) |
| nfvPPA | 0 (0.0) | 0 (0.0) | 0 (0.0) | | 43 (1.5) | 0 (0.0) |
| CBS | 1 (0.1) | 4 (0.3) | 0 (0.0) | | 116 (4.0) | 0 (0.0) |
| PSP | 0 (0.0) | 0 (0.0) | 0 (0.0) | | 102 (3.5) | 2 (0.2) |
| DLB | 4 (0.2) | 5 (0.3) | 6 (0.6) | | 80 (2.8) | 18 (1.5) |
| Mixed/Unspecified Dementia | 116 (6.2) | 0 (0.0) | 14 (1.5) | | 0 (0.0) | 311 (25.3) |
| Psych | 0 (0.0) | 0 (0.0) | 52 (5.5) | | 0 (0.0) | 0 (0.0) |
| Other | 10 (0.5) | 280 (19.4) | 59 (6.2) | | 678 (23.5) | 11 (0.9) |
| Missing/Does not meet criteria | 19 (1.0) | 17 (1.2) | 25 (2.6) | | 73 (2.5) | 187 (15.2) |
| UDS-M+ | 0.08 (0.99) | 0.36 (0.61) | | 0.08 (0.83) | -0.67 (1.09) | -0.02 (1.04) |
| Presents mean (standard deviation) or count (percent) of demographic variables, diagnoses, and cognitive scores. Data are presented after combining the Developmental cohort and DVCID.  The “Mixed/Unspecified Dementia” category included participants with multiple degenerative conditions (e.g., vascular dementia and Alzheimer’s disease) and individuals with dementia due to unknown/unlisted causes. The “Other” category of clinical diagnoses included a range of conditions with low sample sizes including unspecified frontotemporal lobar degeneration, multiple system atrophy, and unspecified primary progressive aphasia. The “Missing/Does not meet criteria” category includes those who were missing diagnoses and those who may have cognitive impairment due to an unknown/unspecified condition.  Abbreviations: **AVLT**: Rey Auditory Verbal Learning Test; **CVLT-II**: California Verbal Learning Test-II Standard Form; **CVLT-SF**: California Verbal Learning Test-II Short Form; **HVLT**: Hopkins Verbal Learning Test Revised; **CERAD**: Consortium to Establish a Registry for Alzheimer's Disease Word List Memory Test; **AD:** Alzheimer’s disease dementia syndrome; **bvFTD**: behavioral variant frontotemporal dementia; **CDR-sb**: Clinical Dementia Rating Scale Sum of Boxes; **CDR-g**: Clinical Dementia Rating Scale Global Score; **DLB**: Dementia with Lewy bodies; **MCI**: Mild Cognitive Impairment; **nfvPPA**: nonfluent-primary progressive aphasia; **PPA:** primary progressive aphasia; **CBS**: Corticobasal syndrome; **PSP**: Progressive supranuclear palsy**; Psych**: Suspected psychiatry primary etiology; **UDS-M+**: Uniform Data Set Memory plus List-Learning score; **DVCID**: Diverse Vascular Contributions to Cognitive Impairment and Dementia. | | | | | | |

| Supplementary Table 2: Fit statistics for the UDS-M+ models | | | |
| --- | --- | --- | --- |
|  | RMSEA | CFI | TLI |
| AVLT [Initial Model] | 0.044 | 0.997 | 0.996 |
| CVLT-II | 0.047 | 0.977 | 0.982 |
| HVLT | 0.052 | 0.991 | 0.993 |
| CVLT-SF | 0.032 | 0.997 | 0.998 |
| CERAD | 0.027 | 0.991 | 0.993 |
| Presents fit statistics for each of the five list-learning models. Each fit statistic was interpreted as excellent or adequate based on thresholds commonly used in other harmonization studies (Mukherjee et al. 2023): RMSEA: <0.05 or <0.08; CFI: >0.90 or ≥ 0.95; TLI: >0.90 or ≥ 0.95. **Abbreviations**: **AVLT**: Rey Auditory Verbal Learning Test; **CERAD**: Consortium to Establish a Registry for Alzheimer's Disease; **CVLT-II:** California Verbal Learning Test-II Standard Form; **CVLT-SF**: CVLT-II Short Form; **HVLT**: Hopkins Verbal Learning Test; **RMSEA**: Root mean square error of approximation; **CFI**: Comparative fit index; **TLI**: Tucker-Lewis index. | | | |

| Supplementary Table 3: Standardized loadings for the AVLT-anchored model building and the CERAD-anchored model building | | | | | | | |
| --- | --- | --- | --- | --- | --- | --- | --- |
|  | **UDS-M+**  **Loading** | | | **Secondary Factor**  **Loading** | | | |
| **Item** | | **AVLT-**  **Anchored** | **CERAD- Anchored** | **AVLT- Anchored** | **CERAD- Anchored** | **Secondary Factor Label** | |
| MoCA Cued Recall | | 0.437 | 0.466 | 0.403 | 0.427 | MoCA | |
| MoCA Recognition Hits | | 0.533 | 0.505 | 0.871 | 0.873 | MoCA | |
| MoCA Free Recall | | 0.872 | 0.930 | 0.053 | 0.229 | MoCA | |
| Craft Immediate Paraphrased | | 0.933 | 0.965 | 0.561 | 0.637 | Craft Story | |
| Craft Delayed Paraphrased | | 0.969 | 0.976 | 0.533 | 0.523 | Craft Story | |
| Craft Immediate Verbatim | | 0.913 | 0.980 | 0.547 | 0.672 | Craft Story | |
| Craft Delayed Verbatim | | 0.933 | 0.964 | 0.535 | 0.483 | Craft Story | |
| AVLT Delayed Recall | | 0.910 | 0.914 | 0.313 | 0.370 | AVLT | |
| AVLT Immediate Trial 1 | | 0.763 | 0.790 | 0.385 | 0.359 | AVLT | |
| AVLT Immediate Trial 2 | | 0.894 | 0.892 | 0.445 | 0.430 | AVLT | |
| AVLT Immediate Trial 3 | | 0.943 | 0.933 | 0.466 | 0.485 | AVLT | |
| AVLT Immediate Trial 4 | | 0.948 | 0.950 | 0.408 | 0.473 | AVLT | |
| AVLT Immediate Trial 5 | | 0.921 | 0.954 | 0.371 | 0.431 | AVLT | |
| CVLT-II Immediate Trial 1 | | 0.866 | 0.874 | 0.535 | 0.538 | CVLT-II | |
| CVLT-II Immediate Trial 2 | | 0.955 | 0.958 | 0.572 | 0.578 | CVLT-II | |
| CVLT-II Immediate Trial 3 | | 0.950 | 0.954 | 0.392 | 0.404 | CVLT-II | |
| CVLT-II Immediate Trial 4 | | 0.962 | 0.965 | 0.324 | 0.340 | CVLT-II | |
| CVLT-II Immediate Trial 5 | | 0.950 | 0.954 | 0.310 | 0.326 | CVLT-II | |
| HVLT Delayed Recall | | 0.881 | 0.891 | 0.330 | 0.380 | HVLT | |
| HVLT Immediate Trial 1 | | 0.814 | 0.832 | 0.443 | 0.469 | HVLT | |
| HVLT Immediate Trial 2 | | 0.934 | 0.940 | 0.544 | 0.567 | HVLT | |
| HVLT Immediate Trial 3 | | 0.950 | 0.956 | 0.492 | 0.525 | HVLT | |
| HVLT Recognition Hits | | 0.743 | 0.762 | 0.266 | 0.308 | HVLT | |
| CVLT-SF Immediate Trial 1 | | 0.795 | 0.747 | 0.607 | 0.640 | CVLT-SF | |
| CVLT-SF Immediate Trial 2 | | 0.914 | 0.889 | 0.609 | 0.657 | CVLT-SF | |
| CVLT-SF Immediate Trial 3 | | 0.931 | 0.913 | 0.524 | 0.582 | CVLT-SF | |
| CVLT-SF Immediate Trial 4 | | 0.919 | 0.899 | 0.480 | 0.540 | CVLT-SF | |
| CVLT-SF Delayed Recall | | 0.928 | 0.894 | 0.130 | 0.230 | CVLT-SF | |
| CVLT-SF Recognition Hits | | 0.612 | 0.560 | 0.090 | 0.159 | CVLT-SF | |
| CERAD Delayed Recall | | 0.888 | 0.919 | 0.534 | 0.537 | CERAD | |
| CERAD Immediate Trial 1 | | 0.679 | 0.742 | 0.586 | 0.594 | CERAD | |
| CERAD Immediate Trial 2 | | 0.890 | 0.921 | 0.718 | 0.721 | CERAD | |
| CERAD Immediate Trial 3 | | 0.884 | 0.914 | 0.642 | 0.643 | CERAD | |
| CERAD Recognition Hits | | 0.604 | 0.664 | 0.225 | 0.210 | CERAD | |
| CERAD Recognition False Positives | | 0.427 | 0.496 | 0.381 | 0.416 | CERAD | |
| MoCA Immediate Recall | | 0.821 | 0.825 | --- | --- | --- | |
| MoCA Orientation Month | | 0.861 | 0.835 | --- | --- | --- | |
| MoCA Orientation Year | | 0.874 | 0.862 | --- | --- | --- | |
| MoCA Orientation Place | | 0.833 | 0.778 | --- | --- | | --- |
| Benson Recall | | 0.819 | 0.825 | --- | --- | | --- |
| Benson Recognition | | 0.660 | 0.730 | --- | --- | | --- |
| CVLT-II Delayed Recall | | 0.991 | 0.993 | --- | --- | | --- |
| CVLT-II Recognition Hits | | 0.839 | 0.851 | --- | --- | | --- |
| CVLT-II Recognition False Positives | | 0.894 | 0.902 | --- | --- | | --- |
| HVLT Recognition False Positives | | 0.648 | 0.677 | --- | --- | | --- |
| CVLT-SF Recognition False Positives | | 0.574 | 0.557 | --- | --- | | --- |
| Presents standardized factor loadings for each item on the primary memory factor (UDS-M+ Loading) and, where applicable, on a secondary factor (Secondary Factor Loading). Each secondary factor is defined in the final column, “Secondary Factor Label.” Items with a “---” in the Secondary Factor Loading column loaded only on the memory factor. Their loadings on the secondary factors were removed due to poor model fit and/or weak magnitudes. Data are presented for a set of models that used the AVLT as the first model (AVLT-Anchored) and for a set of models that used the CERAD as the first model (CERAD-Anchored).  Abbreviations: **AVLT**: Rey Auditory Verbal Learning Test; **CVLT-II**: California Verbal Learning Test-II Standard Form; **CVLT-SF**: California Verbal Learning Test-II Short Form; **HVLT**: Hopkins Verbal Learning Test Revised; **CERAD**: Consortium to Establish a Registry for Alzheimer's Disease Word List Memory Test. | | | | | | | |

| Supplementary Table 4: Standardized thresholds for the AVLT-anchored model building and the CERAD-anchored model building | | | |
| --- | --- | --- | --- |
|  |  | Thresholds | |
| Item | Binned Value | AVLT-Anchored | CERAD-Anchored |
| CVLT-II Immediate Trial 1 | 1 | -1.338 | -1.243 |
| CVLT-II Immediate Trial 1 | 2 | -0.805 | -0.727 |
| CVLT-II Immediate Trial 1 | 3 | -0.287 | -0.226 |
| CVLT-II Immediate Trial 1 | 4 | 0.159 | 0.205 |
| CVLT-II Immediate Trial 1 | 5 | 0.596 | 0.628 |
| CVLT-II Immediate Trial 1 | 6 | 0.905 | 0.928 |
| CVLT-II Immediate Trial 1 | 7 | 1.180 | 1.194 |
| CVLT-II Immediate Trial 1 | 8 | 1.454 | 1.459 |
| CVLT-II Immediate Trial 1 | 9 | 1.684 | 1.681 |
| CVLT-II Immediate Trial 2 | 1 | -0.909 | -0.817 |
| CVLT-II Immediate Trial 2 | 2 | -0.589 | -0.509 |
| CVLT-II Immediate Trial 2 | 3 | -0.263 | -0.196 |
| CVLT-II Immediate Trial 2 | 4 | 0.015 | 0.071 |
| CVLT-II Immediate Trial 2 | 5 | 0.245 | 0.292 |
| CVLT-II Immediate Trial 2 | 6 | 0.495 | 0.532 |
| CVLT-II Immediate Trial 2 | 7 | 0.707 | 0.736 |
| CVLT-II Immediate Trial 2 | 8 | 1.001 | 1.019 |
| CVLT-II Immediate Trial 2 | 9 | 1.202 | 1.212 |
| CVLT-II Immediate Trial 3 | 1 | -1.044 | -0.946 |
| CVLT-II Immediate Trial 3 | 2 | -0.743 | -0.658 |
| CVLT-II Immediate Trial 3 | 3 | -0.562 | -0.483 |
| CVLT-II Immediate Trial 3 | 4 | -0.129 | -0.067 |
| CVLT-II Immediate Trial 3 | 5 | 0.091 | 0.144 |
| CVLT-II Immediate Trial 3 | 6 | 0.290 | 0.336 |
| CVLT-II Immediate Trial 3 | 7 | 0.707 | 0.736 |
| CVLT-II Immediate Trial 4 | 1 | -0.817 | -0.728 |
| CVLT-II Immediate Trial 4 | 2 | -0.663 | -0.581 |
| CVLT-II Immediate Trial 4 | 3 | -0.491 | -0.415 |
| CVLT-II Immediate Trial 4 | 4 | -0.283 | -0.215 |
| CVLT-II Immediate Trial 4 | 5 | -0.087 | -0.027 |
| CVLT-II Immediate Trial 4 | 6 | 0.142 | 0.193 |
| CVLT-II Immediate Trial 4 | 7 | 0.333 | 0.378 |
| CVLT-II Immediate Trial 4 | 8 | 0.556 | 0.591 |
| CVLT-II Immediate Trial 5 | 1 | -1.016 | -0.921 |
| CVLT-II Immediate Trial 5 | 2 | -0.841 | -0.752 |
| CVLT-II Immediate Trial 5 | 3 | -0.670 | -0.587 |
| CVLT-II Immediate Trial 5 | 4 | -0.387 | -0.315 |
| CVLT-II Immediate Trial 5 | 5 | -0.189 | -0.126 |
| CVLT-II Immediate Trial 5 | 6 | 0.031 | 0.087 |
| CVLT-II Immediate Trial 5 | 7 | 0.202 | 0.251 |
| CVLT-II Immediate Trial 5 | 8 | 0.415 | 0.456 |
| CVLT-II Delayed Recall | 1 | -0.631 | -0.534 |
| CVLT-II Delayed Recall | 2 | -0.522 | -0.431 |
| CVLT-II Delayed Recall | 3 | -0.315 | -0.235 |
| CVLT-II Delayed Recall | 4 | -0.245 | -0.169 |
| CVLT-II Delayed Recall | 5 | -0.015 | 0.049 |
| CVLT-II Delayed Recall | 6 | 0.081 | 0.140 |
| CVLT-II Delayed Recall | 7 | 0.323 | 0.369 |
| CVLT-II Delayed Recall | 8 | 0.443 | 0.483 |
| CVLT-II Delayed Recall | 9 | 0.560 | 0.594 |
| CVLT-II Recognition Hits | 1 | -1.283 | -1.184 |
| CVLT-II Recognition Hits | 2 | -1.126 | -1.033 |
| CVLT-II Recognition Hits | 3 | -0.942 | -0.856 |
| CVLT-II Recognition Hits | 4 | -0.811 | -0.729 |
| CVLT-II Recognition Hits | 5 | -0.563 | -0.490 |
| CVLT-II Recognition Hits | 6 | -0.291 | -0.228 |
| CVLT-II Recognition Hits | 7 | 0.044 | 0.095 |
| CVLT-II Recognition False Positives | 1 | -0.919 | -0.826 |
| CVLT-II Recognition False Positives | 2 | -0.832 | -0.742 |
| CVLT-II Recognition False Positives | 3 | -0.763 | -0.676 |
| CVLT-II Recognition False Positives | 4 | -0.616 | -0.535 |
| CVLT-II Recognition False Positives | 5 | -0.509 | -0.432 |
| CVLT-II Recognition False Positives | 6 | -0.353 | -0.283 |
| CVLT-II Recognition False Positives | 7 | -0.299 | -0.231 |
| CVLT-II Recognition False Positives | 8 | -0.144 | -0.082 |
| CVLT-II Recognition False Positives | 9 | -0.031 | 0.026 |
| CVLT-SF Immediate Trial 1 | 1 | -2.347 | -2.411 |
| CVLT-SF Immediate Trial 1 | 2 | -1.832 | -1.852 |
| CVLT-SF Immediate Trial 1 | 3 | -1.257 | -1.228 |
| CVLT-SF Immediate Trial 1 | 4 | -0.633 | -0.549 |
| CVLT-SF Immediate Trial 1 | 5 | 0.024 | 0.165 |
| CVLT-SF Immediate Trial 1 | 6 | 0.743 | 0.945 |
| CVLT-SF Immediate Trial 1 | 7 | 1.461 | 1.725 |
| CVLT-SF Immediate Trial 2 | 1 | -2.831 | -3.019 |
| CVLT-SF Immediate Trial 2 | 2 | -2.350 | -2.478 |
| CVLT-SF Immediate Trial 2 | 3 | -1.863 | -1.930 |
| CVLT-SF Immediate Trial 2 | 4 | -1.375 | -1.380 |
| CVLT-SF Immediate Trial 2 | 5 | -0.813 | -0.750 |
| CVLT-SF Immediate Trial 2 | 6 | -0.247 | -0.115 |
| CVLT-SF Immediate Trial 2 | 7 | 0.302 | 0.503 |
| CVLT-SF Immediate Trial 2 | 8 | 0.856 | 1.125 |
| CVLT-SF Immediate Trial 3 | 1 | -2.779 | -2.950 |
| CVLT-SF Immediate Trial 3 | 2 | -2.411 | -2.539 |
| CVLT-SF Immediate Trial 3 | 3 | -1.968 | -2.042 |
| CVLT-SF Immediate Trial 3 | 4 | -1.510 | -1.530 |
| CVLT-SF Immediate Trial 3 | 5 | -1.056 | -1.022 |
| CVLT-SF Immediate Trial 3 | 6 | -0.590 | -0.499 |
| CVLT-SF Immediate Trial 3 | 7 | -0.112 | 0.036 |
| CVLT-SF Immediate Trial 3 | 8 | 0.365 | 0.570 |
| CVLT-SF Immediate Trial 4 | 1 | -2.704 | -2.852 |
| CVLT-SF Immediate Trial 4 | 2 | -2.378 | -2.490 |
| CVLT-SF Immediate Trial 4 | 3 | -1.979 | -2.046 |
| CVLT-SF Immediate Trial 4 | 4 | -1.575 | -1.596 |
| CVLT-SF Immediate Trial 4 | 5 | -1.162 | -1.136 |
| CVLT-SF Immediate Trial 4 | 6 | -0.755 | -0.685 |
| CVLT-SF Immediate Trial 4 | 7 | -0.305 | -0.183 |
| CVLT-SF Immediate Trial 4 | 8 | 0.130 | 0.301 |
| CVLT-SF Delayed Recall | 1 | -1.128 | -1.077 |
| CVLT-SF Delayed Recall | 2 | -0.927 | -0.858 |
| CVLT-SF Delayed Recall | 3 | -0.753 | -0.668 |
| CVLT-SF Delayed Recall | 4 | -0.549 | -0.446 |
| CVLT-SF Delayed Recall | 5 | -0.351 | -0.230 |
| CVLT-SF Delayed Recall | 6 | -0.114 | 0.028 |
| CVLT-SF Delayed Recall | 7 | 0.116 | 0.278 |
| CVLT-SF Delayed Recall | 8 | 0.427 | 0.617 |
| CVLT-SF Recognition Hits | 1 | -3.626 | -3.668 |
| CVLT-SF Recognition Hits | 2 | -1.841 | -1.814 |
| CVLT-SF Recognition Hits | 3 | -1.281 | -1.232 |
| CVLT-SF Recognition Hits | 4 | -0.767 | -0.698 |
| CVLT-SF Recognition False Positives | 1 | -3.534 | -3.555 |
| CVLT-SF Recognition False Positives | 2 | -1.657 | -1.628 |
| CVLT-SF Recognition False Positives | 3 | -1.123 | -1.082 |
| CVLT-SF Recognition False Positives | 4 | -0.715 | -0.663 |
| CERAD Immediate Trial 1 | 1 | -1.659 | -1.585 |
| CERAD Immediate Trial 1 | 2 | -1.080 | -1.051 |
| CERAD Immediate Trial 1 | 3 | -0.437 | -0.463 |
| CERAD Immediate Trial 1 | 4 | 0.155 | 0.087 |
| CERAD Immediate Trial 1 | 5 | 0.748 | 0.629 |
| CERAD Immediate Trial 1 | 6 | 1.331 | 1.165 |
| CERAD Immediate Trial 1 | 7 | 1.915 | 1.723 |
| CERAD Immediate Trial 2 | 1 | -2.504 | -2.248 |
| CERAD Immediate Trial 2 | 2 | -2.150 | -1.965 |
| CERAD Immediate Trial 2 | 3 | -1.501 | -1.379 |
| CERAD Immediate Trial 2 | 4 | -0.963 | -0.911 |
| CERAD Immediate Trial 2 | 5 | -0.374 | -0.395 |
| CERAD Immediate Trial 2 | 6 | 0.106 | 0.014 |
| CERAD Immediate Trial 2 | 7 | 0.661 | 0.508 |
| CERAD Immediate Trial 2 | 8 | 1.248 | 1.017 |
| CERAD Immediate Trial 2 | 9 | 1.935 | 1.600 |
| CERAD Immediate Trial 3 | 1 | -2.603 | -2.392 |
| CERAD Immediate Trial 3 | 2 | -2.116 | -1.928 |
| CERAD Immediate Trial 3 | 3 | -1.654 | -1.519 |
| CERAD Immediate Trial 3 | 4 | -1.245 | -1.163 |
| CERAD Immediate Trial 3 | 5 | -0.804 | -0.780 |
| CERAD Immediate Trial 3 | 6 | -0.312 | -0.343 |
| CERAD Immediate Trial 3 | 7 | 0.179 | 0.080 |
| CERAD Immediate Trial 3 | 8 | 0.710 | 0.538 |
| CERAD Immediate Trial 3 | 9 | 1.246 | 1.007 |
| CERAD Delayed Recall | 1 | -0.916 | -0.863 |
| CERAD Delayed Recall | 2 | -0.669 | -0.658 |
| CERAD Delayed Recall | 3 | -0.423 | -0.442 |
| CERAD Delayed Recall | 4 | -0.153 | -0.205 |
| CERAD Delayed Recall | 5 | 0.140 | 0.046 |
| CERAD Delayed Recall | 6 | 0.461 | 0.325 |
| CERAD Delayed Recall | 7 | 0.777 | 0.591 |
| CERAD Delayed Recall | 8 | 1.158 | 0.925 |
| CERAD Delayed Recall | 9 | 1.607 | 1.315 |
| CERAD Recognition Hits | 1 | -1.251 | -1.214 |
| CERAD Recognition Hits | 2 | -0.939 | -0.917 |
| CERAD Recognition Hits | 3 | -0.664 | -0.656 |
| CERAD Recognition Hits | 4 | -0.277 | -0.307 |
| CERAD Recognition False Positives | 1 | -1.408 | -1.413 |
| CERAD Recognition False Positives | 2 | -1.117 | -1.118 |
| CERAD Recognition False Positives | 3 | -0.866 | -0.886 |
| CERAD Recognition False Positives | 4 | -0.524 | -0.554 |
| AVLT Immediate Trial 1 | 1 | -1.739 | -1.780 |
| AVLT Immediate Trial 1 | 2 | -1.219 | -1.311 |
| AVLT Immediate Trial 1 | 3 | -0.710 | -0.851 |
| AVLT Immediate Trial 1 | 4 | -0.157 | -0.340 |
| AVLT Immediate Trial 1 | 5 | 0.365 | 0.173 |
| AVLT Immediate Trial 1 | 6 | 0.938 | 0.744 |
| AVLT Immediate Trial 1 | 7 | 1.415 | 1.246 |
| AVLT Immediate Trial 1 | 8 | 1.872 | 1.658 |
| AVLT Immediate Trial 1 | 9 | 2.207 | 2.025 |
| AVLT Immediate Trial 2 | 1 | -2.246 | -2.301 |
| AVLT Immediate Trial 2 | 2 | -1.795 | -1.801 |
| AVLT Immediate Trial 2 | 3 | -1.017 | -1.120 |
| AVLT Immediate Trial 2 | 4 | -0.677 | -0.805 |
| AVLT Immediate Trial 2 | 5 | -0.336 | -0.505 |
| AVLT Immediate Trial 2 | 6 | 0.467 | 0.302 |
| AVLT Immediate Trial 2 | 7 | 0.842 | 0.684 |
| AVLT Immediate Trial 2 | 8 | 1.229 | 1.064 |
| AVLT Immediate Trial 2 | 9 | 2.039 | 1.838 |
| AVLT Immediate Trial 3 | 1 | -2.358 | -2.414 |
| AVLT Immediate Trial 3 | 2 | -1.966 | -2.072 |
| AVLT Immediate Trial 3 | 3 | -1.255 | -1.337 |
| AVLT Immediate Trial 3 | 4 | -0.955 | -1.083 |
| AVLT Immediate Trial 3 | 5 | -0.678 | -0.842 |
| AVLT Immediate Trial 3 | 6 | -0.143 | -0.327 |
| AVLT Immediate Trial 3 | 7 | 0.162 | -0.025 |
| AVLT Immediate Trial 3 | 8 | 0.534 | 0.377 |
| AVLT Immediate Trial 3 | 9 | 1.282 | 1.124 |
| AVLT Immediate Trial 4 | 1 | -2.339 | -2.454 |
| AVLT Immediate Trial 4 | 2 | -2.016 | -2.134 |
| AVLT Immediate Trial 4 | 3 | -1.318 | -1.404 |
| AVLT Immediate Trial 4 | 4 | -1.048 | -1.178 |
| AVLT Immediate Trial 4 | 5 | -0.832 | -0.968 |
| AVLT Immediate Trial 4 | 6 | -0.421 | -0.600 |
| AVLT Immediate Trial 4 | 7 | -0.171 | -0.349 |
| AVLT Immediate Trial 4 | 8 | 0.101 | -0.059 |
| AVLT Immediate Trial 4 | 9 | 0.808 | 0.661 |
| AVLT Immediate Trial 5 | 1 | -2.345 | -2.564 |
| AVLT Immediate Trial 5 | 2 | -1.995 | -2.120 |
| AVLT Immediate Trial 5 | 3 | -1.378 | -1.464 |
| AVLT Immediate Trial 5 | 4 | -1.137 | -1.273 |
| AVLT Immediate Trial 5 | 5 | -0.926 | -1.082 |
| AVLT Immediate Trial 5 | 6 | -0.522 | -0.716 |
| AVLT Immediate Trial 5 | 7 | -0.326 | -0.531 |
| AVLT Immediate Trial 5 | 8 | -0.087 | -0.269 |
| AVLT Immediate Trial 5 | 9 | 0.552 | 0.393 |
| AVLT Delayed Recall | 1 | -0.990 | -1.150 |
| AVLT Delayed Recall | 2 | -0.822 | -0.965 |
| AVLT Delayed Recall | 3 | -0.530 | -0.704 |
| AVLT Delayed Recall | 4 | -0.384 | -0.545 |
| AVLT Delayed Recall | 5 | -0.210 | -0.372 |
| AVLT Delayed Recall | 6 | 0.149 | 0.017 |
| AVLT Delayed Recall | 7 | 0.381 | 0.277 |
| AVLT Delayed Recall | 8 | 0.627 | 0.530 |
| AVLT Delayed Recall | 9 | 1.194 | 1.090 |
| HVLT Immediate Trial 1 | 1 | -1.987 | -1.990 |
| HVLT Immediate Trial 1 | 2 | -1.665 | -1.682 |
| HVLT Immediate Trial 1 | 3 | -1.234 | -1.270 |
| HVLT Immediate Trial 1 | 4 | -0.802 | -0.857 |
| HVLT Immediate Trial 1 | 5 | -0.388 | -0.463 |
| HVLT Immediate Trial 1 | 6 | 0.025 | -0.068 |
| HVLT Immediate Trial 1 | 7 | 0.548 | 0.431 |
| HVLT Immediate Trial 1 | 8 | 0.965 | 0.830 |
| HVLT Immediate Trial 1 | 9 | 1.671 | 1.504 |
| HVLT Immediate Trial 2 | 1 | -1.595 | -1.602 |
| HVLT Immediate Trial 2 | 2 | -1.196 | -1.227 |
| HVLT Immediate Trial 2 | 3 | -0.776 | -0.833 |
| HVLT Immediate Trial 2 | 4 | -0.456 | -0.532 |
| HVLT Immediate Trial 2 | 5 | -0.112 | -0.209 |
| HVLT Immediate Trial 2 | 6 | 0.259 | 0.140 |
| HVLT Immediate Trial 2 | 7 | 0.674 | 0.529 |
| HVLT Immediate Trial 3 | 1 | -1.773 | -1.771 |
| HVLT Immediate Trial 3 | 2 | -1.256 | -1.285 |
| HVLT Immediate Trial 3 | 3 | -0.963 | -1.010 |
| HVLT Immediate Trial 3 | 4 | -0.601 | -0.670 |
| HVLT Immediate Trial 3 | 5 | -0.379 | -0.462 |
| HVLT Immediate Trial 3 | 6 | -0.111 | -0.209 |
| HVLT Immediate Trial 3 | 7 | 0.252 | 0.131 |
| HVLT Delayed Recall | 1 | -0.392 | -0.471 |
| HVLT Delayed Recall | 2 | -0.323 | -0.406 |
| HVLT Delayed Recall | 3 | -0.273 | -0.359 |
| HVLT Delayed Recall | 4 | -0.176 | -0.267 |
| HVLT Delayed Recall | 5 | -0.036 | -0.133 |
| HVLT Delayed Recall | 6 | 0.106 | 0.002 |
| HVLT Delayed Recall | 7 | 0.284 | 0.172 |
| HVLT Delayed Recall | 8 | 0.524 | 0.400 |
| HVLT Delayed Recall | 9 | 0.777 | 0.642 |
| HVLT Recognition Hits | 1 | -1.429 | -1.464 |
| HVLT Recognition Hits | 2 | -1.091 | -1.137 |
| HVLT Recognition Hits | 3 | -0.812 | -0.868 |
| HVLT Recognition Hits | 4 | -0.369 | -0.440 |
| HVLT Recognition False Positives | 1 | -1.377 | -1.398 |
| HVLT Recognition False Positives | 2 | -1.163 | -1.192 |
| HVLT Recognition False Positives | 3 | -0.931 | -0.968 |
| HVLT Recognition False Positives | 4 | -0.593 | -0.644 |
| HVLT Recognition False Positives | 5 | -0.174 | -0.241 |
| Craft Immediate Paraphrased | 1 | -1.904 | -1.873 |
| Craft Immediate Paraphrased | 2 | -1.412 | -1.539 |
| Craft Immediate Paraphrased | 3 | -1.057 | -1.233 |
| Craft Immediate Paraphrased | 4 | -0.642 | -0.744 |
| Craft Immediate Paraphrased | 5 | -0.385 | -0.487 |
| Craft Immediate Paraphrased | 6 | -0.024 | -0.127 |
| Craft Immediate Paraphrased | 7 | 0.548 | 0.487 |
| Craft Immediate Paraphrased | 8 | 1.080 | 0.972 |
| Craft Immediate Paraphrased | 9 | 1.670 | 1.610 |
| Craft Delayed Paraphrased | 1 | -1.207 | -1.077 |
| Craft Delayed Paraphrased | 2 | -0.926 | -0.880 |
| Craft Delayed Paraphrased | 3 | -0.718 | -0.732 |
| Craft Delayed Paraphrased | 4 | -0.425 | -0.353 |
| Craft Delayed Paraphrased | 5 | -0.142 | -0.180 |
| Craft Delayed Paraphrased | 6 | 0.191 | 0.091 |
| Craft Delayed Paraphrased | 7 | 0.521 | 0.455 |
| Craft Delayed Paraphrased | 8 | 1.260 | 1.060 |
| Craft Delayed Paraphrased | 9 | 1.862 | 1.622 |
| Craft Immediate Verbatim | 1 | -1.779 | -2.043 |
| Craft Immediate Verbatim | 2 | -1.158 | -1.342 |
| Craft Immediate Verbatim | 3 | -0.781 | -0.975 |
| Craft Immediate Verbatim | 4 | -0.397 | -0.618 |
| Craft Immediate Verbatim | 5 | 0.046 | -0.041 |
| Craft Immediate Verbatim | 6 | 0.440 | 0.298 |
| Craft Immediate Verbatim | 7 | 1.021 | 0.935 |
| Craft Immediate Verbatim | 8 | 1.465 | 1.435 |
| Craft Immediate Verbatim | 9 | 2.210 | 2.229 |
| Craft Delayed Verbatim | 1 | -1.151 | -1.039 |
| Craft Delayed Verbatim | 2 | -0.921 | -0.942 |
| Craft Delayed Verbatim | 3 | -0.690 | -0.741 |
| Craft Delayed Verbatim | 4 | -0.437 | -0.514 |
| Craft Delayed Verbatim | 5 | -0.241 | -0.319 |
| Craft Delayed Verbatim | 6 | -0.003 | -0.144 |
| Craft Delayed Verbatim | 7 | 0.253 | 0.037 |
| Craft Delayed Verbatim | 8 | 0.660 | 0.356 |
| Craft Delayed Verbatim | 9 | 1.022 | 0.630 |
| MoCA Free Recall | 1 | -0.574 | -0.642 |
| MoCA Free Recall | 2 | -0.354 | -0.431 |
| MoCA Free Recall | 3 | -0.029 | -0.157 |
| MoCA Free Recall | 4 | 0.395 | 0.207 |
| MoCA Cued Recall | 1 | -0.675 | -0.834 |
| MoCA Cued Recall | 2 | 0.357 | 0.130 |
| MoCA Cued Recall | 3 | 0.810 | 0.625 |
| MoCA Cued Recall | 4 | 0.993 | 0.776 |
| MoCA Recognition Hits | 1 | -2.907 | -2.835 |
| MoCA Recognition Hits | 2 | -0.449 | -0.546 |
| MoCA Recognition Hits | 3 | 0.617 | 0.481 |
| MoCA Recognition Hits | 4 | 1.234 | 1.025 |
| MoCA Immediate Recall | 1 | -1.530 | -1.593 |
| MoCA Immediate Recall | 2 | -1.353 | -1.457 |
| MoCA Immediate Recall | 3 | -1.194 | -1.272 |
| MoCA Immediate Recall | 4 | -0.976 | -0.958 |
| MoCA Immediate Recall | 5 | -0.652 | -0.535 |
| MoCA Orientation Month | 1 | -1.508 | -1.526 |
| MoCA Orientation Year | 1 | -1.444 | -1.228 |
| MoCA Orientation Place | 1 | -1.173 | -1.000 |
| Benson Recall | 1 | -1.130 | -1.144 |
| Benson Recall | 2 | -0.931 | -0.948 |
| Benson Recall | 3 | -0.861 | -0.857 |
| Benson Recall | 4 | -0.617 | -0.618 |
| Benson Recall | 5 | -0.492 | -0.397 |
| Benson Recall | 6 | -0.202 | -0.030 |
| Benson Recall | 7 | -0.043 | 0.204 |
| Benson Recall | 8 | 0.469 | 0.628 |
| Benson Recall | 9 | 0.768 | 0.977 |
| Benson Recognition | 1 | -0.767 | -0.747 |
| Presents standardized thresholds for items delineated by which model was used as the anchor model (AVLT vs CERAD).  Abbreviations: **AVLT**: Rey Auditory Verbal Learning Test; **CVLT-II**: California Verbal Learning Test-II Standard Form; **CVLT-SF**: California Verbal Learning Test-II Short Form; **HVLT**: Hopkins Verbal Learning Test Revised; **CERAD**: Consortium to Establish a Registry for Alzheimer's Disease Word List Memory Test. | | | |

| Supplementary Table 5: Validation analyses by UDS-M+ version | | |
| --- | --- | --- |
|  | **Mplus** | **MIRT** |
| Logical Memory Immediate (r) | 0.753 [0.72, 0.78] | 0.756 [0.73, 0.78] |
| Logical Memory Delayed (r) | 0.770 [0.74, 0.80] | 0.771 [0.74, 0.79] |
| TabCAT Favorites (r) | 0.694 [0.62, 0.76] | 0.692 [0.61, 0.76] |
| ptau-217 (r) | -0.530 [-0.75, -0.20] | -0.530 [-0.75, -0.21] |
| MTL volume (β) | 0.205 [0.11, 0.30] | 0.259 [0.15, 0.37] |
| Presents a subset of analyses using the Mplus- and MIRT-based versions of the UDS-M+. Each column provides a Pearson correlation (r) or linear regression beta coefficient (β) and 95% confidence intervals. All analyses were significant at *p* < 0.001. The medial temporal lobe (MTL) analysis covaries for age, total intracranial volume, and CDR sum of boxes. | | |

Figures

Supplementary Figure 1: Test information curves by list-learning task group

Presents test information plots for the UDS-M+ derived from the UDS-M+ model within the Developmental cohort. Gray lines are placed at 3.33 (corresponding to a reliability of 70%) and 10 (corresponding to a reliability of 90%). Each line was formed by summing the item information for the items within the corresponding test-specific model.

Abbreviations: **AVLT**: Rey Auditory Verbal Learning Test; **CVLT-II**: California Verbal Learning Test-II Standard Form; **CVLT-SF**: California Verbal Learning Test-II Short Form; **HVLT**: Hopkins Verbal Learning Test Revised; **CERAD**: Consortium to Establish a Registry for Alzheimer's Disease Word List Memory Test; **UDS-M+**: Uniform Data Set Memory plus List-Learning score.

Supplementary Figure 2: Comparison of factor scores derived from a CERAD-first and AVLT-first models

Presents a scatterplot showing the association between a factor score derived from a set of models with the AVLT as the anchor model (X-axis) and a factor score derived from a set of models with the CERAD as the anchor model (Y-axis). Each dot represents a single individual who had both scores. The blue line is a nonlinear best-fit line.

Supplementary Figure 3: Density plots by diagnosis and clinical severity

**B.**

**A.**

Presents UDS-M+ score distribution by (A) diagnosis and (B) CDR global scores.

Abbreviations: **UDS-M+**: Uniform Data Set Memory plus List-Learning score; **CU**: cognitively unimpaired; **MCI**: mild cognitive impairment; **AD**: Alzheimer’s disease dementia syndrome; **CDR**: Clinical Dementia Rating Scale.

Supplemental Figure 4: Association between a Mplus- and MIRT-based UDS-M+

Provides the Pearson correlation between Mplus- and MIRT-based versions of the UDS-M+ within the Developmental sample (r=0.99; 95% CI: 0.99, 0.99; p<0.001).

Chalmers, R. Philip. 2012. “Mirt: A Multidimensional Item Response Theory Package for the R Environment.” *Journal of Statistical Software* 48:1–29.

Chang, Winston, Joe Cheng, Joseph J. Allaire, Carson Sievert, Barret Schloerke, Yihui Xie, Jeff Allen, Jonathan McPherson, Alan Dipert, and Barbara Borges. 2012. “Shiny: Web Application Framework for R.” *(No Title)*.

Mukherjee, Shubhabrata, Seo-Eun Choi, Michael L. Lee, Phoebe Scollard, Emily H. Trittschuh, Jesse Mez, Andrew J. Saykin, Laura E. Gibbons, R. Elizabeth Sanders, Andrew F. Zaman, Merilee A. Teylan, Walter A. Kukull, Lisa L. Barnes, David A. Bennett, Andrea Z. Lacroix, Eric B. Larson, Michael Cuccaro, Shannon Mercado, Logan Dumitrescu, Timothy J. Hohman, and Paul K. Crane. 2023. “Cognitive Domain Harmonization and Cocalibration in Studies of Older Adults.” *Neuropsychology* 37(4):409–23. doi:10.1037/neu0000835.
